# Shedding Light on Solid Sorbents: Evaluation of Supported Potassium Carbonate Particle Size and Its Effect on CO_2_ Capture from Air

**DOI:** 10.1021/acs.iecr.2c01508

**Published:** 2022-09-10

**Authors:** Nazila Masoud, Victorien Clement, Tomas van Haasterecht, Marlene Führer, Jan P. Hofmann, Johannes Hendrik Bitter

**Affiliations:** † Biobased Chemistry and Technology, Wageningen University, P.O. Box 17, 6700AA Wageningen, The Netherlands; ‡ Laboratory for Inorganic Materials and Catalysis, Department of Chemical Engineering and Chemistry, Eindhoven University of Technology, P.O. Box 513, 5600MB Eindhoven, The Netherlands; § Surface Science Laboratory, Department of Materials and Earth Sciences, Technical University of Darmstadt, Otto-Berndt-Strasse 3, 64287 Darmstadt, Germany

## Abstract

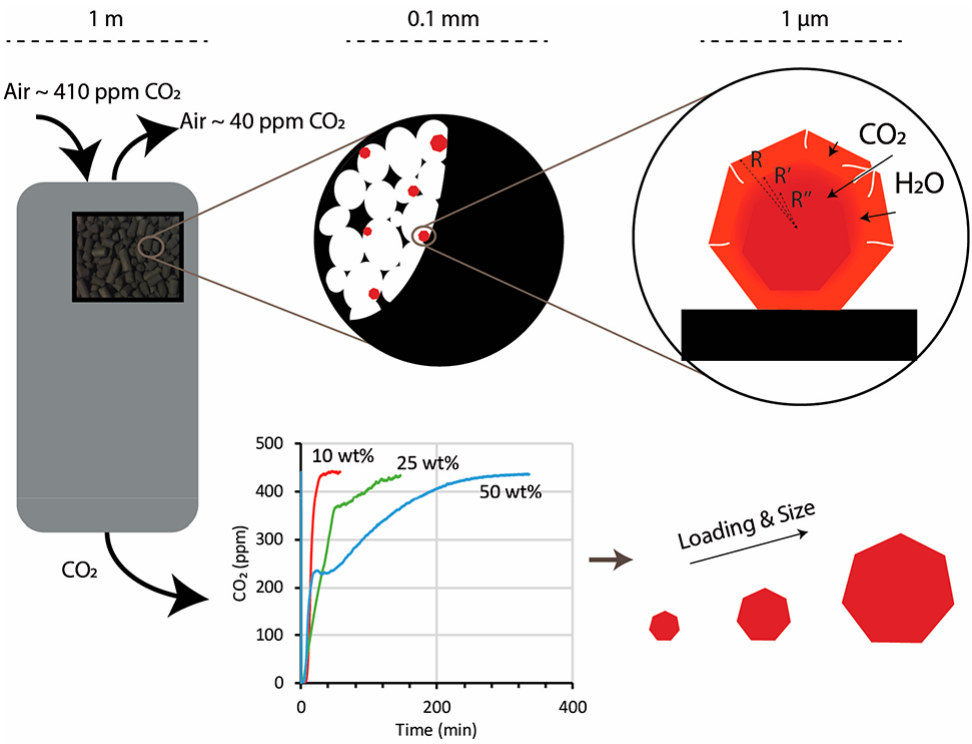

Solid sorbents are essential for developing technologies that directly capture CO_2_ from air. In solid sorbents, metal oxides and/or alkali metal carbonates such as potassium carbonate (K_2_CO_3_) are promising active components owing to their high thermal stability, low cost, and ability to chemisorb the CO_2_ present at low concentrations in air. However, this chemisorption process is likely limited by internal diffusion of CO_2_ into the bulk of K_2_CO_3_. Therefore, the size of the K_2_CO_3_ particles is expected to be an important factor in determining the kinetics of the sorption process during CO_2_ capture. To date, the effects of particle size on supported K_2_CO_3_ sorbents are unknown mainly because particle sizes cannot be unambiguously determined. Here, we show that by using a series of techniques, the size of supported K_2_CO_3_ particles can be established. We prepared size-tuned carbon-supported K_2_CO_3_ particles by tuning the K_2_CO_3_ loading. We further used melting point depression of K_2_CO_3_ particles to collectively estimate the average K_2_CO_3_ particle sizes. Using these obtained average particle sizes, we show that the particle size critically affects the efficiency of the sorbent in CO_2_ capture from air and directly affects the kinetics of CO_2_ sorption as well as the energy input needed for the desorption step. By evaluating the mechanisms involved in the diffusion of CO_2_ and H_2_O into K_2_CO_3_ particles, we relate the microscopic characteristics of sorbents to their macroscopic performance, which is of interest for industrial-scale CO_2_ capture from air.

## 1 Introduction

In heterogeneous catalysis, it is well-known that the size of the active component can affect a catalyst’s performance via geometric and electronic effects.^1,2^ For instance, smaller supported metal nanoparticles have more low-coordinate corner and edge sites and fewer planar sites than larger particles. These two types of sites show different catalytic activities. For instance, corner and edge sites are the most active for CO oxidation by gold catalysts; thus, smaller particles are desired for this application.^1^ Conversely planar sites are more active for propylene epoxidation by silver catalysts, so larger particles are desired.^3^ The reason for this difference is that the low-coordinate sites have more dangling bonds than planar sites and therefore exhibit different reactivity. Moreover, below 2 nm, the band structure of the particle changes to a more discrete orbital structure that alters the electronic properties and thus the reactivity of the particles. Though the effect of particle size on catalysts has been well studied, little is known about the particle size effect on the performance of a similar category of materials, namely, solid sorbents, in which a solute reacts with the sorbent’s active component that is dispersed over a support.

Solid sorbents are promising materials for CO_2_ capture from air or flue gas.^4−6^ Here, we focus on CO_2_ capture from air, where a solid sorbent typically functions in a temperature-dependent process.^7,8^ Capture takes place at low temperatures, and the sorbent is regenerated by heating, after which the desorbed CO_2_ is collected for further use (carbon capture and utilization) or storage (carbon capture and storage). For an efficient process, it is essential that the sorbent has a high capture capacity and a fast capture rate. Desorption must take place at a low temperature to minimize the required energy input.

In general, CO_2_ can be adsorbed on a solid via physisorption or chemisorption. In physisorption, the capture material binds CO_2_ via weak van der Waals interactions. Materials such as microporous activated carbon can capture CO_2_ from highly concentrated sources (10–20 vol %) such as flue gas via physisorption.^9,10^ In chemisorption, materials capture CO_2_ via the formation of chemical bonds. Hence, they are more suitable to capture CO_2_ from low-concentration sources (0.04 vol %) such as air.^11,12^ A broad range of chemisorption materials have been developed for CO_2_ capture from air,^11−14^ such as metal–organic frameworks, immobilized amines, ionic liquids, and basic metal oxides.

**Scheme 1:**
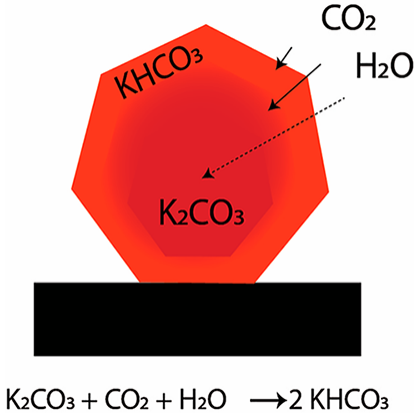
Presentation of a Multilayer Model: CO_2_ and H_2_O Diffuse into the Bulk K_2_CO_3_

Basic metal oxides and alkali metal carbonates such as calcium oxide (CaO), sodium carbonate (Na_2_CO_3_), and potassium carbonate (K_2_CO_3_) are promising solid sorbents.^15−17^ K_2_CO_3_ supported on carbon is especially promising for direct CO_2_ capture from air^18^ because K_2_CO_3_ is thermally stable, is not toxic, is not corrosive, and is inexpensive. It reversibly reacts with CO_2_ in the presence of water at room temperature (RT) to produce potassium bicarbonate (Scheme 1). Complete decomposition of the bicarbonate product can be achieved at temperatures as low as 150 °C, which makes the energy requirements comparable to that of commercially applied amines in the liquid phase for CO_2_ capture from flue gas.^6^

Unlike metal nanoparticles in catalysis, where only the particle outer layer adsorbs the reactants, the sorbent’s active component, such as K_2_CO_3_, potentially absorbs solutes such as CO_2_ throughout the whole particle. Therefore, the sorption processes are best described by multilayer models in which CO_2_ and H_2_O diffuse into and react with the bulk K_2_CO_3_ to form a hydrate and/or bicarbonate (Scheme 1). Hence, the reaction with CO_2_ (carbonation) involves two phases. First, K_2_CO_3_ on the outer layers reacts rapidly. Second, the bulk K_2_CO_3_ reacts in a slower phase that is limited by the diffusion of CO_2_ and H_2_O. That diffusion might be so slow that part of the K_2_CO_3_ in the core may never react.

According to Zhao et al.,^19^ internal diffusion is the rate-determining step during the carbonation of bulk K_2_CO_3_. This (internal) diffusion limitation was also reported for other sorbents, such as Li_2_CO_3_/K_2_CO_3_ and CaO.^20,21^ Therefore, the size of the K_2_CO_3_ particles must be an important factor in determining the capacity and kinetics of the sorbent during CO_2_ capture. It was suggested previously that nanoscale K_2_CO_3_ particles have a higher capacity for the sorbent;^6,22−24^ however, the size was not specified. The main reason for the lack of accurate size data is that no technique has been established to analyze K_2_CO_3_ nanoparticles, especially when they are supported. Therefore, we investigated combinations of techniques to characterize supported alkali metal carbonates such as K_2_CO_3_. Here, we aim to elucidate the size of K_2_CO_3_ particles that are supported on carbon.

We first prepared carbon-supported K_2_CO_3_ particles with different loadings, which were expected to result in different particle sizes. Next, conventional characterization techniques such as X-ray diffraction (XRD), scanning electron microscopy (SEM), and transmission electron microscopy (TEM) were used to establish the particle sizes but achieved limited success. Only TEM provided information about the K_2_CO_3_ particle size. Conversely, X-ray photoelectron spectroscopy (XPS) demonstrated a trend for particle size: The higher the loading was, the larger the K_2_CO_3_ particle size. Melting point depression measurements of K_2_CO_3_, in combination with other techniques, ultimately resulted in the establishment of the average particle sizes in the different samples.

We studied K_2_CO_3_/carbon sorbents for the capture of CO_2_ from air in a fixed-bed flow-through reactor. We observed that the K_2_CO_3_ particle size critically affected the efficiency of the sorbent in CO_2_ capture: The K_2_CO_3_ with the smallest size showed the fastest sorption and required the lowest temperature for CO_2_ desorption. Hence, particle size directly affected the kinetics of the process as well as the required energy input. After the particle size effect was established, we unambiguously established the occurrence of diffusion limitations in the sorption of CO_2_ over supported nanosized K_2_CO_3_.

## 2 Experimental Section

### 2.1 Sample Preparation

Commercially available nonmicroporous carbon (KetjenBlack EC 600JD, AkzoNobel) was used to support K_2_CO_3_. K_2_CO_3_ (Sigma-Aldrich, 99.995% trace metals basis) was deposited on the carbon by incipient wetness impregnation. In a typical preparation, the support (1 g) was dried under vacuum at 200 °C for 2 h. After cooling to RT, it was impregnated with an aqueous K_2_CO_3_ solution (1.7 mL) with an appropriate concentration to prepare 10, 25, and 50 wt % K_2_CO_3_ on the KetjenBlack support. The samples were aged at RT under vacuum for 30 min and then dried in a freeze drier at −80 °C under 0.1 mbar of vacuum for 18 h. The dried samples were collected and named according to their K_2_CO_3_ loading as KB10, KB25, and KB50. At least 4 different batches of each loading were prepared, characterized, and tested for CO_2_ capture from air.

### 2.2 Characterization

Nitrogen physisorption measurements were performed at −196 °C (Micromeritics, TriStar II plus) to determine the BET surface area and porosity of the support and the sorbents.

XRD was carried out at the XPD beamline of the Brazilian Synchrotron Light Laboratory (LNLS) in Campinas, Brazil. The X-ray source was fixed at 8 keV (λ = 1.5498 Å). Powder samples were placed in an in situ high-temperature diffraction chamber (Anton Paar, XRK900) and heated in situ to 200 °C to eliminate the hydrate and bicarbonate forms. Crystallite sizes were determined from the peak broadening at 2θ = 34.1° using the Scherrer equation.

SEM was performed on an FEI Magellan 400 FESEM instrument equipped with an energy dispersive spectroscopy system from Oxford Instruments. For TEM imaging, either the dry powder samples or their diluted mixture with methanol were dispersed on TEM grids. TEM imaging was performed on a JEOL JEM-1400 Plus microscope operated at 120 kV.

XPS was performed on a Thermo Scientific K-alpha spectrometer equipped with a monochromated aluminum Kα source with a photon energy of 1486.6 eV. Samples were handled in air and pressed in a copper powder sample holder without any further fixation. Sample charging was compensated by a flood gun during measurements. Binding energies were calibrated by setting the C 1s component belonging to graphitic sp^2^ carbon to 284.5 eV. Quantitative analysis of the XPS data was performed using Casa XPS software (version 2.3.22PR1.0) based on high-resolution regional scans covering the K 2p and C 1s core levels in the binding energy range of 280–304 eV. For the KB10, KB25, and KB50 samples, the relative XPS intensity of potassium to carbon from the support, (*I*_*p*_/*I*_*s*_)_*exp*_, was obtained by dividing the area of the K 2p peaks by the area of the C 1s sp^2^ peak after correction for the relative sensitivity factors and the mean free path obtained from the included CasaXPS database (Scofield). The (*I*_*p*_/*I*_*s*_)_*exp*_ value was used to estimate the relative size of the supported K_2_CO_3_ particles by using a quantitative XPS analysis model. A summary of the model and calculations is provided in the Supporting Information.

Thermogravimetric analysis (TGA, Mettler-Toledo TGA/DSC1) was used to measure the melting point of the K_2_CO_3_ particles. The samples (∼10–20 mg) were loaded in a 150 μL alumina pan. The change in sample mass and heat flow were recorded during TGA under a flow of air (50 mL/min). Corrections for the buoyancy effect and background heat flow were performed by subtracting the data obtained from a blank experiment performed on the same pan prior to each experiment. The temperature and heat flow were calibrated with reference to a Au sample. The melting point was determined by following the endothermic heat flow at temperatures above 800 °C.

### 2.3 Performance in CO_2_ Capture

CO_2_ capture experiments were performed either via TGA or in a fixed-bed flow-through reactor. In a typical TGA experiment, the sorbent (K_2_CO_3_, KB10, KB25, or KB50, ∼ 5 mg) was placed in a 40 μL aluminum pan. The sorbent was pretreated from RT to 200 °C (ramp 10 °C/min) under flow of dry synthetic air (50 mL/min, zero CO_2_ and H_2_O concentrations) and kept at 200 °C for 30 min. Then, the sorbent was cooled to 60 °C to start the CO_2_ capture experiment. The sorbent was exposed to a flow of air (50 mL/min, ∼430 ppm of CO_2_ and 60 ppm of H_2_O) passing over the sample for 3 h, while the sorbent was kept at 60 °C. To vary the water concentration of the flow, the air was saturated with water vapor at different temperatures. The mass increase due to CO_2_ and water uptake were then recorded.

Experiments in a fixed-bed flow-through reactor were performed in a Micromeritics AutoChem 2920 automated catalyst characterization system. The system was modified in house to involve an air flow with a constant and predetermined humidity. This was achieved by flowing the air constantly through a water vapor generator at a specified temperature. All water-containing lines were placed in a hot zone (110 °C) to stabilize the humidity of the flow.

In a typical experiment, the sorbent (KB10, KB25, or KB50, 50 mg) was placed in a U-shaped quartz plug flow reactor (internal diameter of 9 mm). The bed size was approximately 15 mm, and the gas hourly space velocity was approximately 120000 1/h. Under these conditions, there was no pressure drop over the reactor. Since the KetjenBlack support has a low mechanical stability, the samples were not sieved before use. The sorbent was pretreated from RT to 200 °C (ramp 10 °C/min) under flow of dry He (50 mL/min) and kept at 200 °C for 30 min. Then, the sorbent was cooled to 60 °C to start the CO_2_ capture experiment. The sorbent was exposed to a flow of air (50 mL/min, ∼430 ppm of CO_2_, 3 vol % H_2_O) for a certain time (1, 2.5, 5, or 10 h), while the sorbent was kept at 60 °C. The outlet was analyzed every 1 s by a nondispersive infrared gas detector (LI-840A CO_2_/H_2_O gas analyzer, LI-COR) to record CO_2_ and H_2_O concentrations. CO_2_ breakthrough curves during CO_2_ capture from air were thus obtained. Furthermore, the CO_2_ sorption capacity was obtained by dividing the total moles of CO_2_ uptake by the dry amount of sorbent applied for each test. To calculate the efficiency of CO_2_ uptake, the total moles of CO_2_ uptake were divided by the corresponding moles of K_2_CO_3_ in each test. Here, a 1:1 stochiometric mole ratio of CO_2_ to K_2_CO_3_ was considered. Consecutively, CO_2_ desorption upon heat treatment (10 °C/min up to 200 °C) under a flow of dry N_2_ (50 mL/min) was performed.

## 3 Results and Discussion

### 3.1 Structural Characterizations

To determine the textural properties of the samples and support, nitrogen adsorption–desorption isotherms for the KetjenBlack support, KB10, KB25, and KB50 were analyzed (Supporting Information, Figure S1). From the isotherms, the BET surface area, total pore volume, and micropore volume were obtained and are summarized in Table 1. Upon increasing the K_2_CO_3_ loading, the BET surface area and the total pore volume decreased. This decrease was expected since K_2_CO_3_ is not porous and the support is the only component with a porous structure.

**Table 1:** Summary of the Sample Pore Structures

sample	BET surface area (m^2^/g sample)	total pore volume (mL/g)^a^	expected pore volume (mL/g) if pores are occupied by K_2_CO_3_	micropore volume (mL/g sample)
KetjenBlack	1370	3.16	3.16	0.05
KB10	1210	2.83	2.86	0.04
KB25	950	2.10	2.45	0.03
KB50	700	1.51	1.37	0.02

a Total volume of pores smaller than ∼400 nm width was measured at p/p° of 0.99.

We calculated the expected pore volume if all the pores (maximum size ∼400 nm, measured at p/p° of 0.99) were occupied by K_2_CO_3_ by considering the support pore volume, the K_2_CO_3_ loading of each sample, and the density of K_2_CO_3_ (2.43 g/cm^3^). These values are summarized in Table 1. The total pore volume of the pores was only slightly smaller than the expected pore volume calculated for KB10 and KB25, indicating that the K_2_CO_3_ occupied pores in the support smaller than ∼400 nm in the KB10 and KB25 sorbents. However, the total pore volume of the pores was slightly larger than the expected pore volume calculated for KB50, indicating that K_2_CO_3_ did not fully occupy the pores in KB50. One therefore may assume that the particle size is smaller than 400 nm for KB10 and KB25 and likely larger than 400 nm for KB50. However, determination of the particle size by the limitation of the pores is not explicit because the pores of the KetjenBlack support are not rigid but are prone to expansion and interconnected. Some pores can also be blocked upon deposition of the K_2_CO_3_, and the deviations are not large. Hence, from the physisorption data, one can only assume that KB50 has larger size K_2_CO_3_ particles than the KB10 and KB25 sorbents.

**Figure 1:**
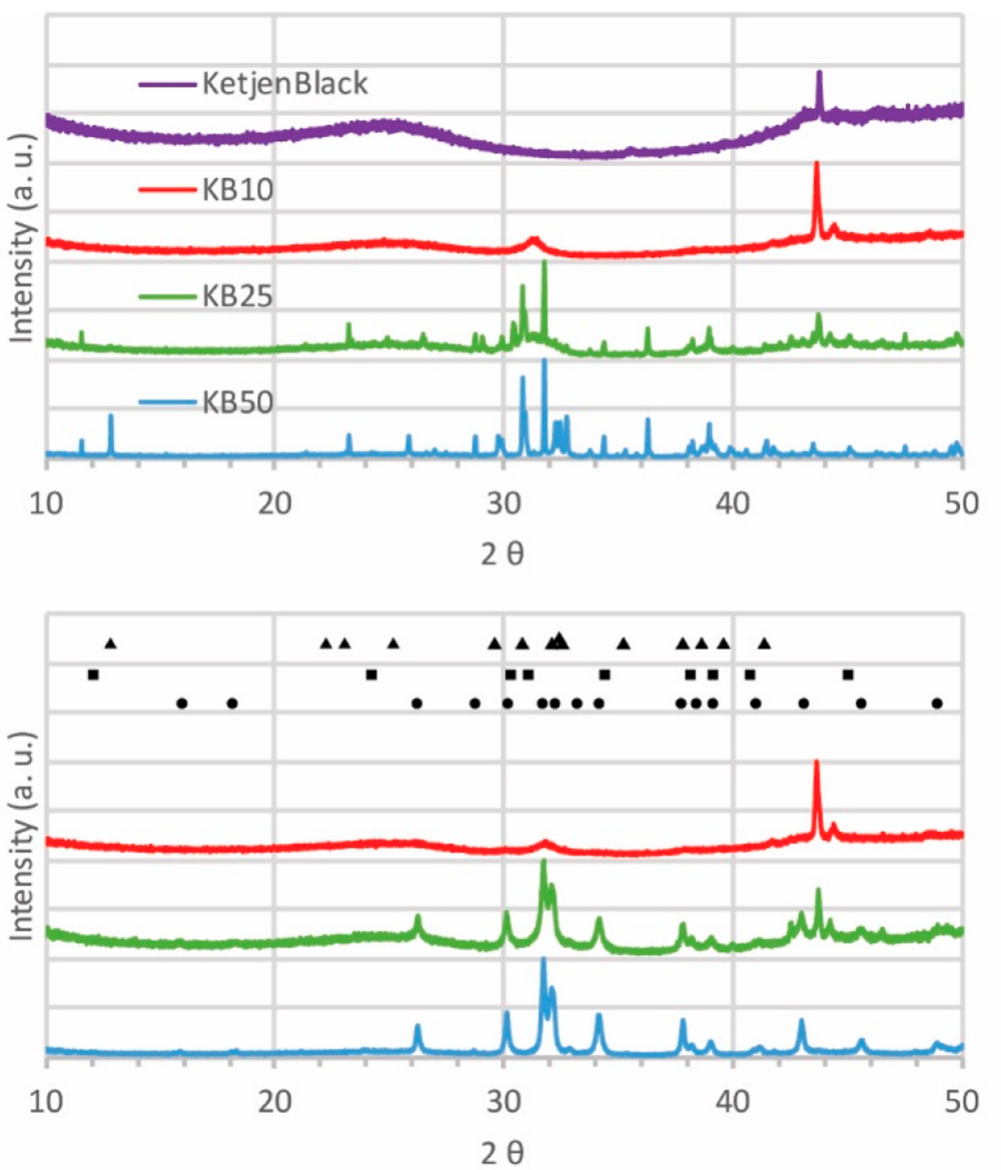
XRD patterns of KB10, KB25, and KB50 before (top) and after (bottom) the samples were heated in situ to 200 °C to eliminate hydrate and bicarbonate forms. References of K_2_CO_3_ (●), hydrate (▲), and bicarbonate (■) are shown on the patterns.

**Figure 2:**
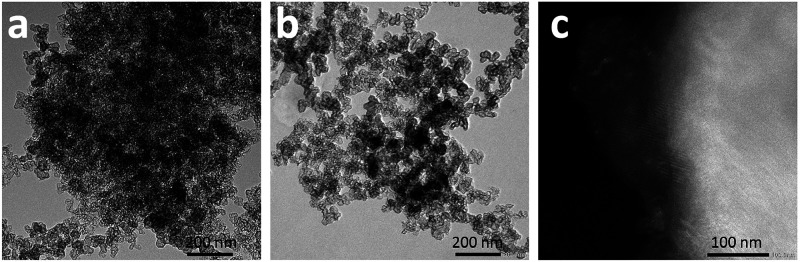
TEM images of KetjenBlack (a) and KB50 (b). Dark-field TEM image of K_2_CO_3_ on a carbon support that shows the presence of a crystalline structure (c).

XRD patterns of the samples (KB10, KB25, and KB50) before and after heating are shown in Figure 1. The combination of different crystal phases, K_2_CO_3_ (reference PDF 87-0730), KHCO_3_ (reference PDF 01-0976) and K_2_CO_3_.1.5H_2_O (reference PDF 11-0655), is visible for the three sorbents before heating. Figure 1 shows that upon in situ heating, all the phases were converted to K_2_CO_3_. From the broadening of the K_2_CO_3_ peak at 34.1°, crystallite sizes of 13, 30, and 36 nm for KB10, KB25, and KB50, respectively, were estimated. Notably, the crystallite size of bulk K_2_CO_3_ was approximately 56 nm, depending on its source and preparation. Therefore, the crystallite sizes of these samples, bulk and supported K_2_CO_3_, are not largely different; however, their particle sizes can still be very different and larger than the crystallite sizes (see below (Figure 2) for TEM where the smallest particle size observed was 140 nm). It is likely that the particle size is larger than the crystallite size, and every particle contains crystallites of different domains in the range of 10–40 nm. Hence, XRD provides an average crystallite size but is not useful to estimate particle sizes.

Next, different techniques were applied to visualize the K_2_CO_3_ particles directly in an attempt to determine their particle sizes. SEM coupled with EDX elemental mapping showed the presence of K_2_CO_3_ on the carbon support (Figure S2). Some very large particles of approximately 18 μm as well as some particles of approximately 0.5 μm were distinguishable in the images. However, nanoparticles were not observed by SEM, although their presence could be inferred from the fact that the elemental mapping indicated the presence of K even when no particles were visible. Extraction of an average particle size was therefore not possible.

TEM images of the carbon support and the K_2_CO_3_/carbon are shown in Figure 2a,b. The images do not differentiate K_2_CO_3_ from the carbon support, as the K_2_CO_3_ and the carbon support have very similar electron densities; hence, they display little contrast in bright-field TEM. Dark-field TEM images of K_2_CO_3_ on the carbon support (Figure 2c) showed some crystalline K_2_CO_3_ structures; however, this technique was not able to clearly distinguish K_2_CO_3_ particles to measure their size.

**Figure 3:**
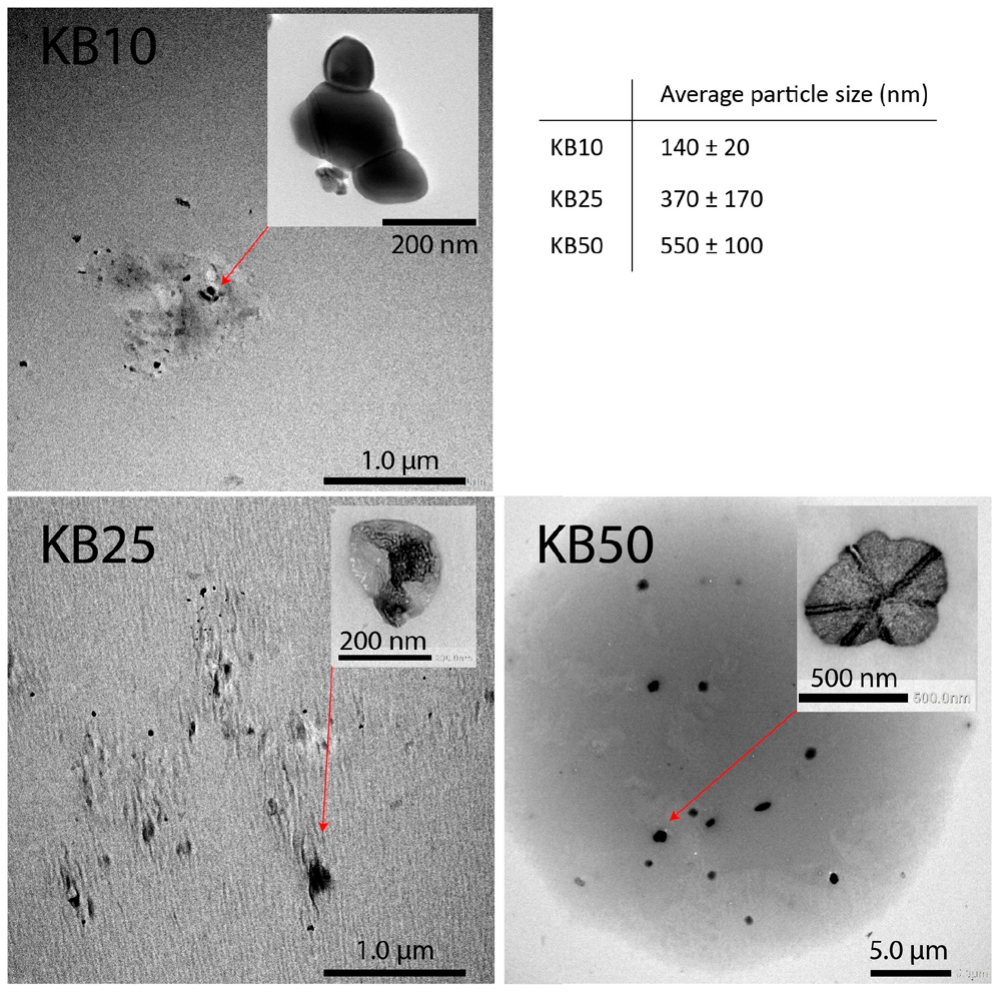
TEM images of KB10, KB25, and KB50 after burning the carbon support in air under TGA conditions.

In an attempt to visualize the K_2_CO_3_ particles by TEM without interference from the carbon support, we removed the carbon by calcining it at 700 °C under air. Since solid K_2_CO_3_ is thermally stable up to its melting point at 895 °C, particles of K_2_CO_3_ were assumed to be intact. TEM imaging (Figure 3) clearly showed particles of K_2_CO_3_ containing different crystalline facets. Approximately 3–10 particles of K_2_CO_3_ could be isolated and measured. Average particle sizes of 140, 370, and 550 nm were estimated for the KB10, KB25, and KB50 samples, respectively, though with a large standard deviation. Hence, TEM imaging suggested a trend for particle size: KB10 < KB25 < KB50.

During imaging, we noticed that K_2_CO_3_ is very sensitive to the electron beam and that the particles changed as soon as they were exposed. TEM images of one K_2_CO_3_ particle, captured over time, show rapid changes in K_2_CO_3_ under the beam (Figure S3). Moreover, the samples seemed to be slightly inhomogeneous. Hence, larger particles could be overlooked during TEM imaging. Consequently, the average particle size is subject to error, although some of the K_2_CO_3_ particles were visualized by TEM. Therefore, there is a need for a bulk technique to determine the average size of the particles. Please note that these supports have mesopores while the supports used in our previous study on support effects has micropores.^25^ We did not observe that limitation gas phase diffusion effects in those microporous supports; thus, we do not expect them to happen in the current mesoporous support

In the following, we estimate the K_2_CO_3_ particle sizes for KB10, KB25, and KB50 via a quantitative XPS analysis model developed by Kerkhof and Moulijn.^26^ A summary of the model, the assumptions used to simplify the model, and the adaptations applied to make it suitable for the case of K_2_CO_3_ supported on carbon are explained in the Supporting Information. In summary, the model quantitatively correlates the signal ratio between an element of the supported metal/metal oxide and an element of the support to the dispersion of the metal/metal oxide on that support. Since smaller particles have higher dispersion and XPS is a surface-sensitive technique, a higher signal ratio for metal(oxide)/support is an indicator of smaller particles. Here, we used the K/C ratio to obtain a K_2_CO_3_ particle size. Particle sizes of 3, 7, and 30 nm were estimated by applying the model to KB10, KB25, and KB50, respectively.

**Scheme 2:**
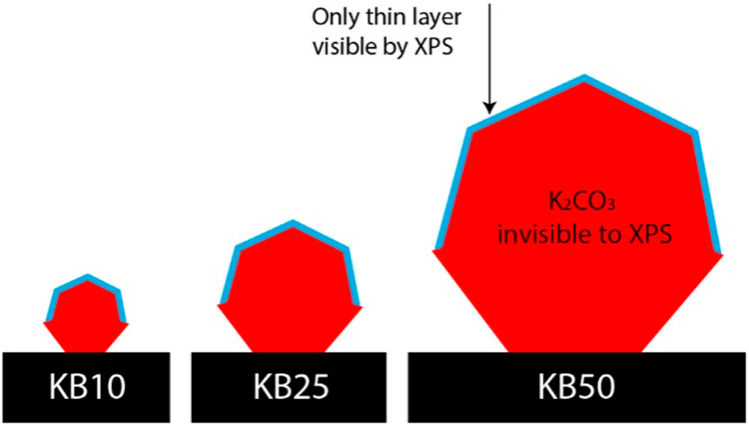
Presentation of K_2_CO_3_ Particles with Different Sizes and the Portion That Is Visible by XPS

The sizes determined by the Kerkhof–Moulijn method are in a different range than expected based on the TEM images of K_2_CO_3_ particles (3–30 nm vs 100–550 nm). This discrepancy is because the particles are too large for the XPS method; thus, the majority of K_2_CO_3_ is invisible by XPS. Scheme 2 illustrates the portion of K_2_CO_3_ particles that are visible by XPS when particles are large and have different sizes. Notably, for simplicity, the particles were considered spheres in the calculation, while they likely have crystalline structures with crystallite faces. In any case, the XPS penetration depth is approximately 3–5 times greater than the inelastic mean free path of the electrons in a medium. The inelastic mean free path of a K 2p electron in K_2_CO_3_ excited by Al Kα radiation is 3.3 nm.^27^ Hence, XPS detected only the skin of the particles with a size no larger than 15 nm (more details on the qualitative XPS can be found in Figures S4 and S5 and Table S1). This skin size is equal for all particles larger than 15 nm. Nevertheless, for smaller particles compared to larger ones, overall, a greater volume of K_2_CO_3_ is visible by XPS. This finding is interpreted as a smaller particle size. Therefore, XPS quantification cannot directly provide the particle sizes but can rank the samples with different K_2_CO_3_ particle sizes based on their volume visible by XPS.

If the model suggests particle sizes of 3, 7, and 30 nm for K_2_CO_3_ in different samples, the visible particle size ratio would be, in order, 1 to 2.4 to 10. The visible volume of the particles can be estimated by raising this particle size ratio to a power of three. Hence, the corresponding volume detected by XPS is 1:14:1070 for KB10, KB25, and KB50. Now, we have a ratio for visible volume in XPS for different particles, which corresponds to the particles’ skin volume that is visible by XPS. One can translate the ratio for visible volume in XPS to a relative particle surface area. This process is possible since the skin size is the same for all the samples (∼15 nm). Hence, the relative surface area of the particles would also be 1:14:1070. We need to extract a ratio for particle size. Hence, a relative particle size was estimated by calculating the square root of this relative surface area: 1:4:30. Accordingly, KB25 is four times larger than KB10, and KB50 is 30 times larger than KB10, although one may consider this approach very speculative.

We further evaluated the melting point depression of the samples. Melting point depression is the phenomenon in which the melting point of a nanoscale material, i.e., metal, metal oxide and salts, decreases upon reduction in the size of the material.^28^ Therefore, melting point depression does provide information on particle size. Melting point depression follows the Gibbs–Thomson relation:^29^
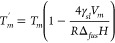
where *T*_*m*_^′^ is the melting temperature of the nanoparticle, *T*_*m*_ is the bulk melting temperature, *γ*_*sl*_ is the solid/liquid surface tension equal to 0.1693 J/m^2^ for K_2_CO_3_,^30,31^*V*_*m*_ is its molar volume equal to 56.87 × 10^–6^ m^3^/mol, *R* is the particle diameter in m, and *Δ*_*fus*_*H* is the K_2_CO_3_ melting molar enthalpy equal to 27.60 × 10^3^ J/mol.

**Figure 4:**
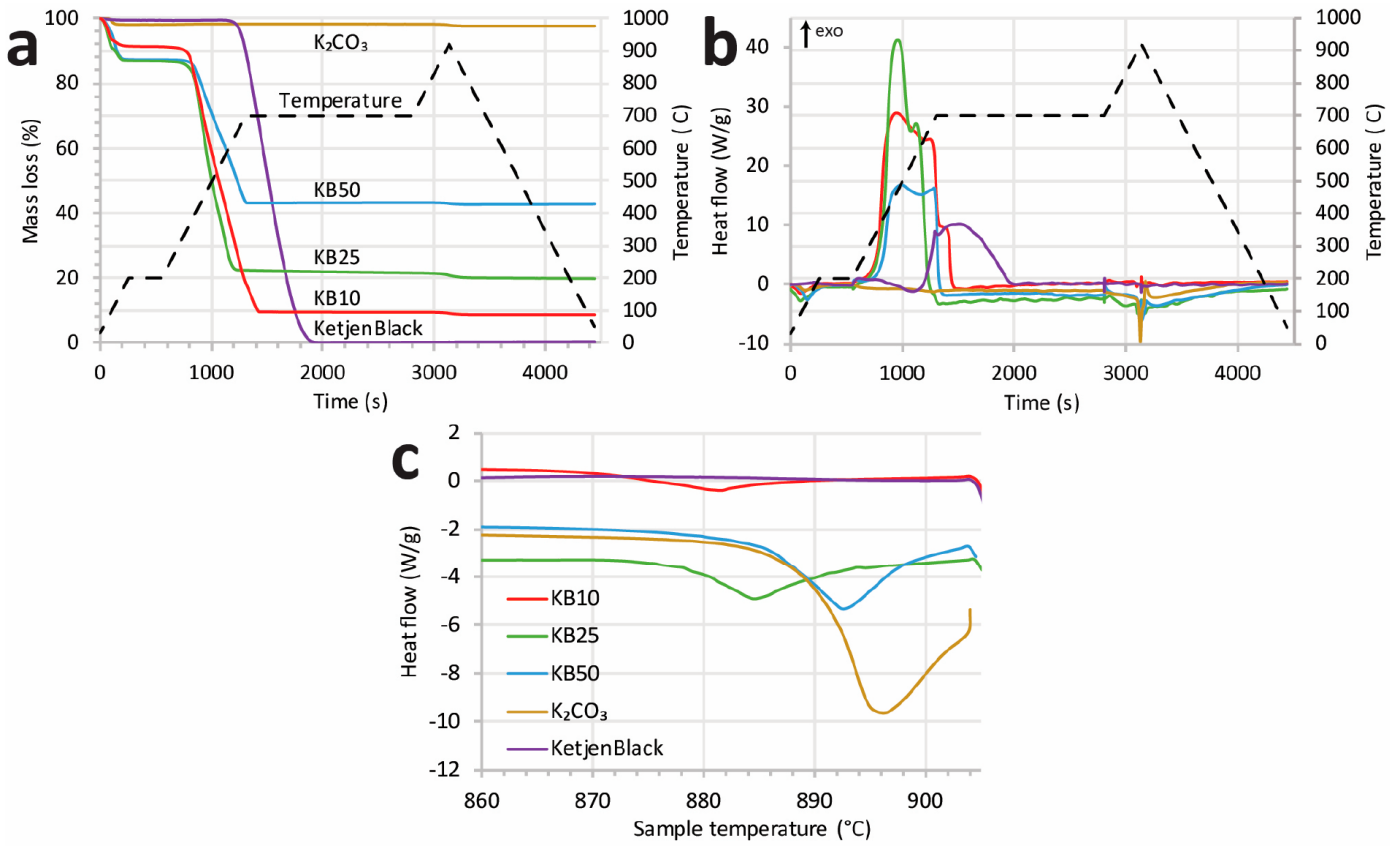
Evolution of the relative mass (a) and heat flow (b and c) with the temperature on KetjenBlack support, bulk K_2_CO_3_, KB10, KB25, and KB50 samples. The samples were heated to 920 °C under flow of synthetic air (50 mL/min, zero CO_2_).

The melting point of the K_2_CO_3_ particles was measured by following the heat flow during TGA under the flow of air (Figure 4). Please note that these experiments had to be performed in air i.e. while burning the carbon. When the experiments were performed in nitrogen (Figures S6 and S7), no unambiguous heat effect was observed most likely because the carbon reacts (is oxidized) with K_2_CO_3_ around its melting point. Figure 4a shows that carbon is burned at temperatures of approximately 700 °C. The mass loss scaled only with the carbon content of the samples; hence, K_2_CO_3_ did not form volatile compounds. Figure 4b shows that by increasing the temperature from 860 to 920 °C, heat consumption due to melting of the K_2_CO_3_ occurred. Figure 4c shows the evolution of heat flow with temperature in the range of 860–920 °C. Bulk K_2_CO_3_ melts at 895 °C. The K_2_CO_3_ in the KB50, KB25, and KB10 samples melted in order at lower temperatures: 894, 888, and 881 °C with ±1 °C variation from triplicate experiments. This result confirms the trend for particle size: KB10 < KB25 < KB50.

By applying the Gibbs–Thomson equation, we estimated a size of 120 ± 20 nm for KB10 and a size of 230 ± 70 nm for KB25. The size for the KB50 sample was estimated to be in the size range of 600–1600 nm, which is similar to the bulk size of approximately 1.5 μm. Though the trend in particle size is clear from the melting point, the absolute particle size has a relatively large error margin because the ±1 °C variation in melting point for these samples lies in a region where a minor change in melting point leads to a considerable change in particle diameter (Figure S8). Nevertheless, from the Gibbs–Thomson equation, one can accurately extract the ratio of particle size for different samples as below:
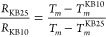
According to the ratio obtained from the melting point depression, KB25 is twice as large as KB10, and KB50 is 14 times larger than KB10.

In summary, TEM, after the carbon support was eliminated, was used to visualize K_2_CO_3_ particles with sizes of 140, 370, 550 nm for KB10, KB25, and KB50, respectively. XPS speculatively suggested a size ratio of 1:4:30, while melting point depression suggested a size ratio of 1:2:14. Melting point depression determined average particle sizes of 120, 230, and ∼1000 nm for KB10, KB25, and KB50, respectively. There are differences in the particle sizes estimated by different techniques. Nevertheless, the trend is clear: The higher the loading is, the larger the K_2_CO_3_ particle size.

### 3.2 Performance in CO_2_ Capture from Air

**Figure 5:**
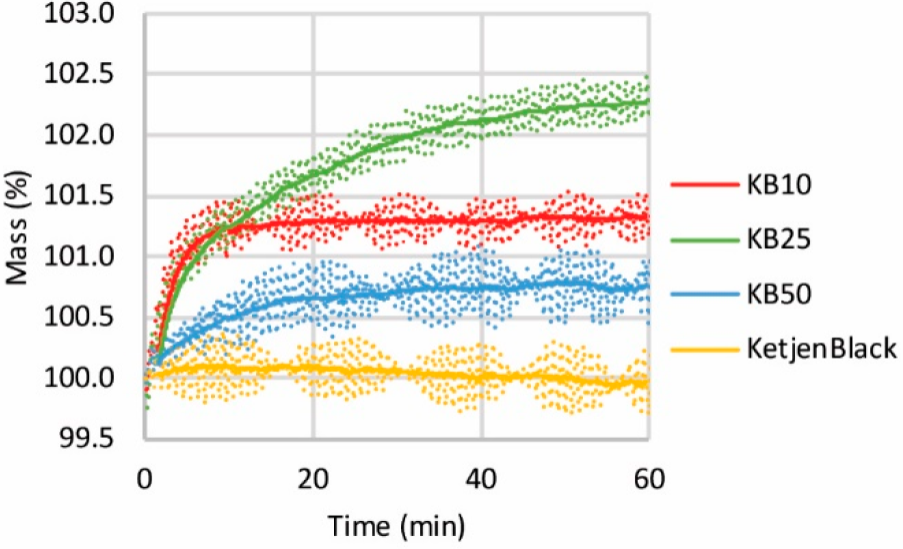
Mass increase due to exposure of the KetjenBlack support, KB10, KB25, and KB50 samples to air (approximately 2 mg of sample), pretreated at 200 °C under 150 mL/min flow of dry synthetic air (no CO_2_) for 1 h, sorption at 60 °C under 150 mL/min flow of air (containing 440 ppm of CO_2_ and 60 ppm of H_2_O).

TGA was used to get an initial idea of the kinetics of the sorbents for CO_2_ capture, as TGA is an often applied technique for this. Please note that our TGA results (and earlier reported results) might suffer from mass transfer limitations. Figure 5 shows the increase in the mass of the sorbents upon exposure to air that contains 440 ppm of CO_2_ and 60 ppm water. A fast initial mass increase occurred for KB10 and KB25. For KB10, the mass increase leveled off to zero, while for KB25, a slow steady mass increase occurred. For KB50, the initial mass increase was slow and leveled off to a very low value.

In these experiments, a low amount of water (60 ppm) was applied to avoid preferential water uptake compared to CO_2_ uptake, which would lead to miscalculation of the capacity of the sorbents in capturing CO_2_. We observed that increasing the water content of the flow led to an increase in the total uptake of the sorbents (Figure S9). It is not trivial to attribute the total mass increase to only CO_2_ uptake, as the formation of a hydrate (K_2_CO_3_·1.5H_2_O) in addition to the bicarbonate (KHCO_3_) is quite possible and likely preferred. It was reported earlier by Jayakumar et al.^32^ that attributing the mass increase in a typical TGA to CO_2_ capture introduces error in the calculation of the sorbent capacity. It is possible that the presence of water and/or CO_2_ at specific concentrations affects the diffusion of the other component as well. Hence, extracting accurate CO_2_ uptake values from TGA data is difficult due to interfering water uptake and/or a low water concentration.

Earlier reports on K_2_CO_3_ for CO_2_ capture were mostly performed via TGA, where unsupported K_2_CO_3_ was exposed to a flow containing CO_2_.^19,32−35^ Therefore, we continue to compare the sorbents in a fixed-bed flow-through reactor in which the changes in CO_2_ concentration are directly measured.

**Figure 6:**
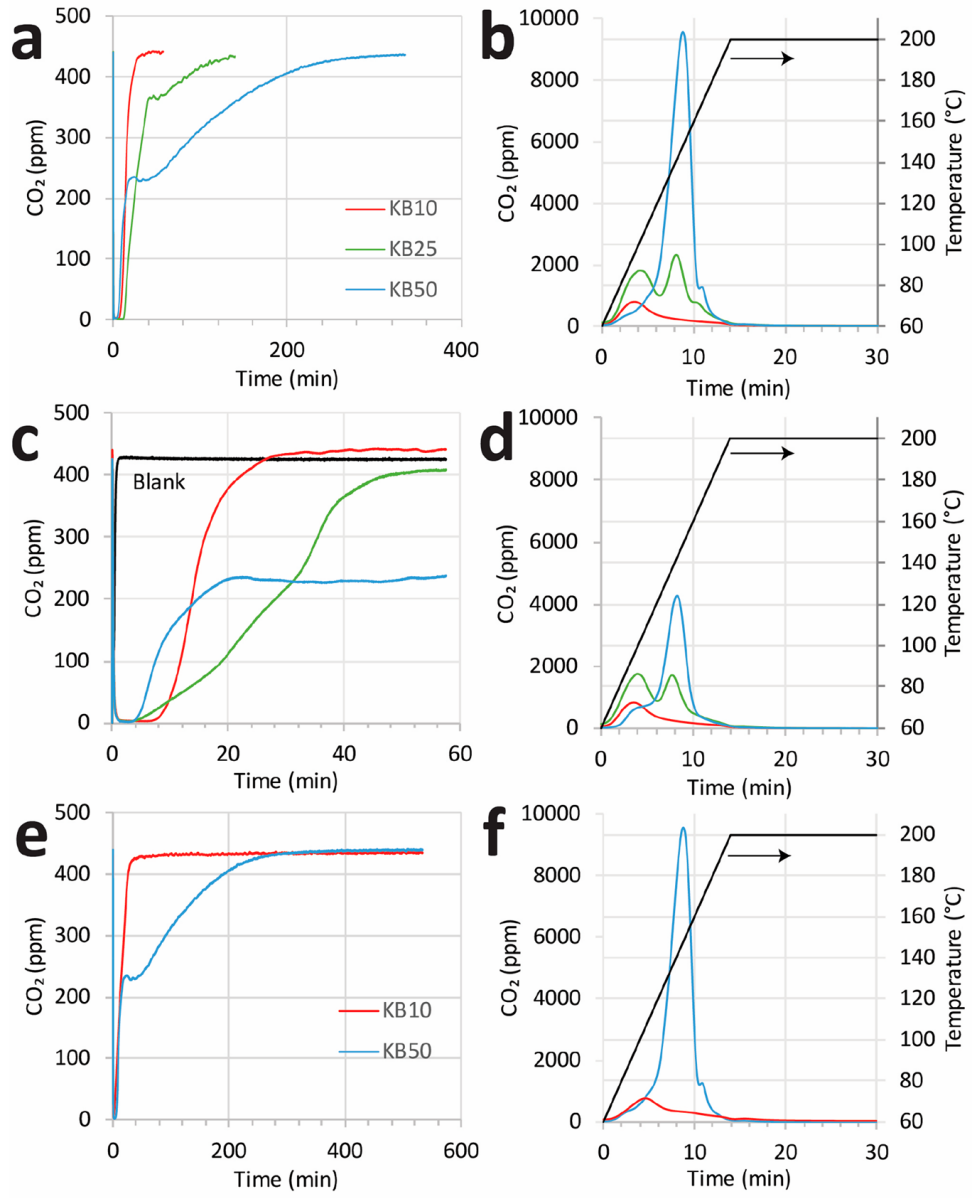
CO_2_ breakthrough curves during CO_2_ capture from air during 5 h (a), 1 h (c), and 10 h (e) of capture. The air passed through different sorbents (KB10, KB25, and KB50, 50 mg), pretreatment RT to 200 °C: 10 °C/min, dwell time of 30 min under flow of dry He (50 mL/min), sorption at 60 °C until reaching the air CO_2_ concentration, flow of air (50 mL/min, 430 ppm of CO_2_), water content: 3 vol % (vapor generated at 25 °C). Consecutive decarbonation upon heat treatment (10 °C/min up to 200 °C) under flow of dry N_2_ (50 mL/min) after 5 h (b), 1 h (d), and 10 h (f) of capture. The CO_2_ concentration was measured by a nondispersive infrared gas detector.

Figure 6a shows the breakthrough of the sorbents in CO_2_ capture. KB10 showed fast sorption and was saturated during the first 20 min of sorption. KB25, however, after an initial fast uptake, continued to capture CO_2_ during 2 h of sorption. KB50, after an initial fast uptake, showed slow and steady uptake during 5 h of sorption. The general trend agrees with the data obtained by TGA. Table 2 summarizes the CO_2_ uptake of the sorbents over time. The highest K_2_CO_3_ loading (KB50 compared with KB25 and KB10) led to the highest total CO_2_ uptake per gram of sorbent in 5 h of capture (1.4 mmol CO_2_/g sorbent). The sorbent with the lowest loading but the smallest K_2_CO_3_ particle size (120 nm for KB10 compared to 230 and 1000 nm for KB25 and KB50) achieved the highest efficiency of CO_2_ capture per amount of K_2_CO_3_ in the shortest time.

**Table 2:** CO_2_ Sorption Capacity of the Samples^a^

sample	total CO_2_ uptake (mmol CO_2_/ g sorbent)	total CO_2_ uptake (mol CO_2_/ mol K_2_CO_3_)	capture time (min)	CO_2_ uptake rate (mol CO_2_/ h·mol K_2_CO_3_)	CO_2_ uptake after 20 min (mol CO_2_/ mol K_2_CO_3_)	CO_2_ uptake after 1 h (mol CO_2_/ mol K_2_CO_3_)	CO_2_ uptake after 10 h (mol CO_2_/ mol K_2_CO_3_)
KB10	0.3	0.5	40	0.7	0.5	0.5	0.5
KB25	0.7	0.4	140	0.2	0.2	0.4	–
KB50	1.4	0.4	335	0.1	0.1	0.2	0.4

a CO_2_ uptake is normalized to the dry amount of the sample.

By examining the breakthrough curves after 1 h of sorption for all three sorbents (Figure 6c), one can see that KB10 with the smallest K_2_CO_3_ particle size showed the fastest sorption compared to KB50 with the largest K_2_CO_3_ size. The CO_2_ uptake rate normalized per mol K_2_CO_3_ was 0.7 mol CO_2_/h for KB10 and 0.1 mol CO_2_/h for KB50. This finding confirms that the sorption follows an initial fast sorption followed by a slower process that occurred only for K_2_CO_3_ with a larger particle size, while for K_2_CO_3_ with a smaller size, the accessible K_2_CO_3_ immediately contributed to the reaction.

Figure 6b shows the consecutive desorption of CO_2_ upon heating the sorbent to 200 °C. Desorption occurred at 100 °C for KB10. The desorption occurred at a higher temperature for KB50 (140 °C). KB25, with an intermediate size, showed two desorption peaks at the temperatures at which desorption occurred for KB10 and KB50. Notably, the decarbonation temperatures for KB10, KB25, and KB50 in the flow unit match the decarbonation temperatures during TGA (Figure S10). The desorption peak height is considerably higher for KB50 than for KB25 and KB10. This shows that KB50 definitely captured more CO_2_ than KB10 and KB25, although at the expense of exposure to air for a longer time and desorption that occurred at higher temperatures.

Consecutive desorption of CO_2_ after 1 h of sorption (Figure 6d) resulted in a lower CO_2_ uptake for KB50 compared to sorption in 5 h. In this case, there was no difference between KB10 and KB25 after sorption for a longer time. This result suggests that diffusion of CO_2_ into K_2_CO_3_ in the KB50 sample with a larger size was limited, since by exposing the KB50 sample to the air flow for a longer time, CO_2_ uptake was increased.

Figure 6e,f shows the CO_2_ breakthrough after 10 h of capture and consecutive desorption of CO_2_ for the KB50 and KB10 sorbents. The figures show that by exposing the KB10 to air for a longer time, CO_2_ capture was not enhanced. Hence, in all three sorbents, regardless of the capture time, at least half of the K_2_CO_3_ remained unreactive. This means that for K_2_CO_3_ with different sizes, diffusion of CO_2_ and water into K_2_CO_3_ is limited to a certain penetration depth, and further diffusion is not possible. Nasiman and Kanoh^23^ reported CO_2_ capture from air under TGA conditions by 60 wt % K_2_CO_3_ in K_2_CO_3_–carbon nanocomposites. From the XRD peak broadening, they estimated a size of approximately 20 nm for K_2_CO_3_ particles. From the provided data, we calculated an efficiency of ∼0.8 mol CO_2_/mol K_2_CO_3_ for that sorbent. These data indicate that with smaller particles, the efficient use of K_2_CO_3_ in a certain period is enhanced. Furthermore, part of the K_2_CO_3_ in the core may never react.

### 3.3 Evaluation of the Diffusion Phenomena

We estimated the CO_2_ penetration depth over time for the KB10, KB25 and KB50 sorbents by considering the estimated size of the K_2_CO_3_ particles obtained by melting point depression and considering the capacity of the sorbents for capturing CO_2_ per mole of the K_2_CO_3_ over time. Every mole of reacted K_2_CO_3_ can be related to an accessible volume in the outer layers. The calculated CO_2_ penetration depths over time for the three sorbents are summarized in Table 3. The penetration depths for KB10 and KB25 are 10 and 20 nm, respectively; however, these values are as high as 80 nm for the KB50 samples. Hence, CO_2_ and H_2_O somehow diffused more into the bulk K_2_CO_3_ in the KB50 sample. Furthermore, one may assume that the CO_2_ and H_2_O diffusion for the KB50 with larger K_2_CO_3_ particles follows a different mechanism than that followed by the KB10 sample with smaller K_2_CO_3_ particles. Different possible diffusion mechanisms are discussed below.

**Table 3:** Calculated CO_2_ Penetration Depth over Time Based on K_2_CO_3_ Particle Size and Capacity of the Samples in CO_2_ Capture

	time of capture (min)	efficiency in CO_2_ capture (mol CO_2_/mol K2CO_3_)	particle diameter (nm)	estimated penetration depth (nm)
KB10	40	0.5	120	10
KB25	140	0.4	230	20
KB50	335	0.4	1000	80

Diffusion of CO_2_ and H_2_O gases into K_2_CO_3_ may follow specific pathways such as microstructures and/or defects that are developed as K_2_CO_3_ particles grow. Generally, the diffusion of ions in solids is dependent on the presence of defects.^36^ It is possible that microstructures and/or defects are developed more as the K_2_CO_3_ particles grow larger, e.g., KB50. The diffusion rate for KB50 was calculated as 3.8 × 10^–7^ cm/min, which is in the range for gas transport in porous material.^37^ This suggests that diffusion via microstructures and/or defects contributed at least in the KB50 sample. Please note that the results up to now indicate that KB50 particles consist of aggregates. This might be the specific microstructure of the KB50 sample which influences its sorption behavior; further study is needed to investigate the exact role of particle size on that.

Moreover, diffusion of CO_2_ and H_2_O gases into K_2_CO_3_ may also follow via an ion conductivity mechanism in which the layer of produced KHCO_3_ acts like a pool of CO_2_ and H_2_O that allows CO_2_ and H_2_O to penetrate into an outer layer of the pool, while inner CO_2_ and H_2_O molecules migrate into the K_2_CO_3_ core. When the KHCO_3_ layer is thickened, the diffusion length is longer; hence, the diffusion of CO_2_ and H_2_O into the core becomes slower according to Fick’s first law of diffusion.^38^ It has been suggested earlier by Luo et al.^39^ that the production of KHCO_3_ can hinder further diffusion of CO_2_ and H_2_O into bulk K_2_CO_3_. Hence, this diffusion mechanism occurs gradually upon formation of additional KHCO_3_ so that, in practice, part of the K_2_CO_3_ in the core may never react. Based on our observation of all three sorbents, regardless of the capture time, at least half of the K_2_CO_3_ remained unreactive. This observation suggests that ion conductivity is an involved mechanism in the diffusion of CO_2_ and H_2_O gases into K_2_CO_3_.

**Figure 7:**
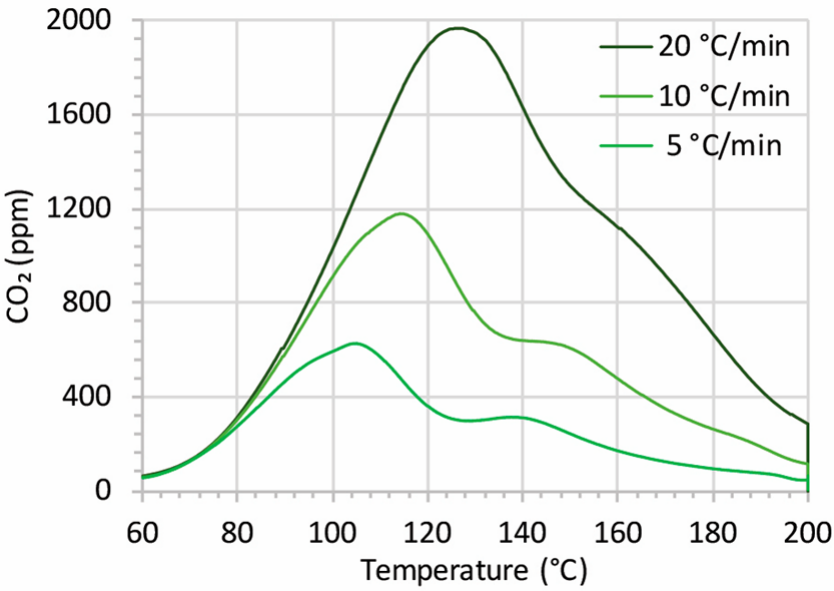
Decarbonation upon heat treatment with different heating rates up to 200 °C under flow of dry N_2_ (50 mL/min) after a 1 h typical capture experiment. The sorbent was KB25.

To further evaluate the diffusion mechanism, we evaluated the decarbonation steps to determine whether they are activated processes. By increasing the heating rate upon decarbonation, one would expect a shift to higher temperatures if the decarbonation is an activated process.^40^Figure 7 shows the desorption of CO_2_ from the KB25 sorbent upon heat treatment. Note that KB25 with an intermediate size showed the dual desorption properties of KB10 and KB50, which means that desorption occurred partially at 100 °C and partially at 140 °C. In all three experiments, the CO_2_ uptake was the same, and upon heat treatment, the captured CO_2_ was fully desorbed. As the heating rate was increased from 5 to 20 °C/min, the peaks due to decarbonation were shifted to higher temperatures. This means that both decarbonation steps were activated processes. The apparent activation energies for both steps were calculated to be 69 and 93 kJ/mol. The basis of the calculation was the Redhead analysis,^40^ and details of the calculation are explained in the Supporting Information.

In the literature, a shrinking core model was used to describe the kinetics of the K_2_CO_3_ carbonation reaction.^19^ According to this model, the reaction takes place only on a surface, dividing the unreacted core from the product shell in one particle, while the core shrinks as the reaction continues. The shrinking core model is valid when the reaction is limited by diffusion of the gases into the core rather than being limited by the reaction kinetics.^41^ According to this model and on the basis of experimental data of CO_2_ capture from flue gas at temperatures of 55–80 °C and a CO_2_ pressure of 0.1 MPa, Zhao et al.^19^ calculated an apparent activation energy of 33.4 kJ/mol for the K_2_CO_3_ carbonation reaction and 99.1 kJ/mol for the diffusion-limited step of the reaction. One can assume that the diffusion-limited step of the carbonation and decarbonation reactions must match. The activation energy of 99.1 kJ/mol for the diffusion-limited step of the reaction in Zhao’s work is close to the activation energy of 93 kJ/mol that we obtained for the second step of decarbonation. These numbers are rather high and might reflect a combination of breaking of chemical bonds and diffusion.^41^ The second step of decarbonation is perhaps fully diffusion limited, as it is more pronounced for KB50 with a larger size of K_2_CO_3_ than for KB10 with a smaller size of K_2_CO_3_. One may note the difference in the reaction conditions of our experiments and the work of Zhao et al.^19^ and note that the experimental data obtained by TGA must be treated carefully due to interfering water effects and possible hydration/dehydration reactions as pointed out by Jayakumar et al.^32^ Nevertheless, we agree with the literature that the K_2_CO_3_ carbonation reaction is diffusion limited, and we suggest a value of 93 kJ/mol for the diffusion-limited step of the carbonation reaction.

Although KB50 with higher K_2_CO_3_ loading showed higher capacity of CO_2_ capture, KB10 with smaller K_2_CO_3_ particles showed higher efficiency of capture. CO_2_ penetrated farther into larger particles but at the expense of longer capture times. This second layer of captured CO_2_ demanded more energy to be desorbed as well. Therefore, the sorbent preparation method must fulfill the criteria of a high loading of K_2_CO_3_ and a small particle size. This is an important consideration for the development of supported solid sorbents, and it is being actively researched in our group. We note here that this reasoning does match with our observations; however, many questions regarding the mechanism remain open like the exact nature of the phases formed during sorption and desorption and the exact kinetics and role of water on that. Another very relevant question is to what extent do all K_2_CO_3_ participate in the sorption process.^42,43^ However, here we focused on the role of particle size and show that there is a particle size effect. More research is needed to fully understand the particle size effect.

## 4 Conclusion

In summary, we prepared a series of carbon-supported K_2_CO_3_ particles with different sizes by varying the K_2_CO_3_ loading. Conventional techniques were not able to decisively determine the average K_2_CO_3_ particle sizes, but melting point depression of the K_2_CO_3_ particles could be used to estimate an average particle size for the three prepared sorbents. This is the first time that the size of supported K_2_CO_3_ nanoparticles has been quantitatively determined and related to performance data of CO_2_ sorption. We further studied the sorbents for the capture of CO_2_ from air in an industrially scalable flow-through setting. The K_2_CO_3_ with the smallest size showed the fastest sorption. The sorbent with the highest K_2_CO_3_ loading showed the highest uptake, though at the expense of a longer time. In this sorbent, with the highest loading and largest K_2_CO_3_ particle size, CO_2_ penetrated farther into the bulk of the particle; however, the internal layers of the captured CO_2_ were desorbed at a higher temperature. We confirmed that desorption is an activated process. The apparent activation energy for the diffusion-limited desorption step was calculated to be 93 kJ/mol. Therefore, the size of the K_2_CO_3_ particles is an important factor in the design of this type of solid sorbents. K_2_CO_3_ particle size critically affected the efficiency of the sorbent in CO_2_ capture relative to the amount of active component being active for sorption, and it directly affects the kinetics of the process as well as the sorbent capacity and energy input for the desorption process. Therefore, we conclude that an ideal sorbent based on supported K_2_CO_3_ must have a high loading of K_2_CO_3_ and a small particle size. This is an important consideration for the development of suitable sorbents for CO_2_ capture from air.

## Supporting Information Available

Nitrogen isotherms of the samples, SEM images and EDX mapping of KB50 and KB25, TEM images of one K_2_CO_3_ particle exposed to beam captured over time, XPS spectra of the samples and quantitative analysis of XPS data including summarized variables applied in the model and the model prediction for K_2_CO_3_ particle sizes, TGA for analysis of melting point depression under nitrogen, description of thermodynamically stable components under nitrogen at high temperatures, the relation of melting point to the K_2_CO_3_ particle size according to the Gibbs–Thomson equation, TGA upon exposure to wet air, TGA during decarbonation, details of the calculation of apparent activation energy for decarbonation steps including variables of the Redhead analysis and Arrhenius plot of the decarbonation steps (PDF)
