# Supplementary material for: Shedding Light on Solid Sorbents: Evaluation of Supported Potassium Carbonate Particle Size and Its Effect on CO2 Capture from Air
Source: Ind Eng Chem Res. 2022 Sep 10;61(38):14211–21. doi: 10.1021/acs.iecr.2c01508 (PMC9524576; doi:10.1021/acs.iecr.2c01508)
Supplement: Supplementary file 1 — ie2c01508_si_001.pdf [file ie2c01508_si_001.pdf]

Supporting information for

Shedding light on solid sorbents: evaluation of supported potassium carbonate particle size and its effect on CO_2 capture from air

Nazila Masoud[1,*], Victorien Clement[1], Tomas van Haasterecht[1], Marlene Führer[1], Jan P. Hofmann[2,3], and Johannes Hendrik Bitter[1,*]

1. Biobased Chemistry and Technology, Wageningen University, P.O. Box 17, 6700AA, Wageningen, The Netherlands

2. Laboratory for Inorganic Materials and Catalysis, Department of Chemical Engineering and Chemistry, Eindhoven University of Technology, P.O. Box 513, 5600MB, Eindhoven, The Netherlands

3. Surface Science Laboratory, Department of Materials and Earth Sciences, Technical University of Darmstadt, Otto-Berndt-Strasse 3, 64287 Darmstadt, Germany

* harry.bitter@wur.nl, nazila.masoud@wur.nl

Table of contents

	Page
**Figure S.1.** Nitrogen isotherms of the samples	2
**Figure S.2.** SEM images and EDX mapping of KB50 and KB25	3
**Figure S.3.** TEM images of one K_2CO_3 particle exposed to beam captured over time	7
**Quantitative XPS analysis**	8
**Scheme S.1.** Schematic presentation of the sample described in the model and supported K_2CO_3 particles on carbon	8
**Figure S.4.** XPS spectra of the samples	9
**Figure S.5.** A general relation showing the percentage of a monolayer promoter on the support	11
**Table S.1.** Summarized variables applied in the model and the model prediction for K_2CO_3 particle sizes..	11
**Figure S.6.** TGA for analysis of melting point depression under nitrogen	12
**Figure S.7.** Thermodynamically stable components under nitrogen at high temperatures	13
**Figure S.8.** The relation of melting point to the K_2CO_3 particle size according to the Gibbs-Thomson equation	14

Figure S.9. TGA upon exposure to wet air 15
Figure S.10. TGA during decarbonation 16
....
Details of calculation of apparent activation energy for decarbonation steps 17
....
Table S.2. Variables of the Redhead analysis 17
Figure S.11. Arrhenius plot of the decarbonation steps 17
Supporting information references 18
....

Figure S.1. Nitrogen adsorption-desorption isotherms for KetjenBlack support, KB10, KB25, and KB50 were measured to establish textural properties of the samples and support

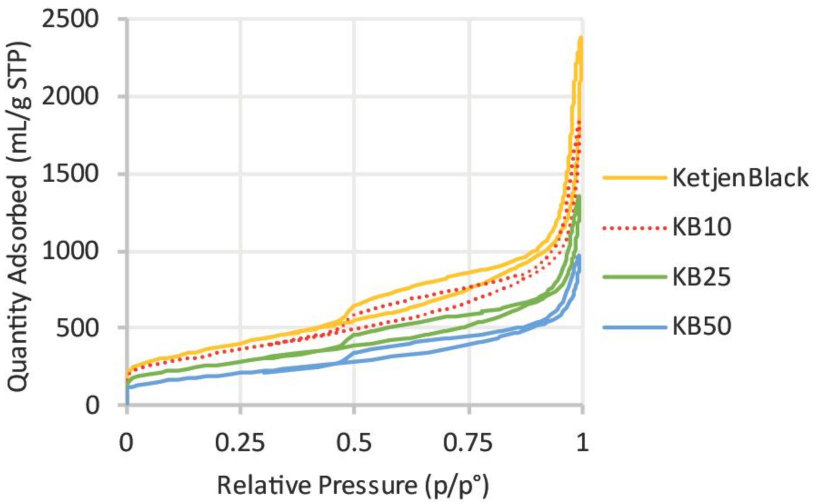

Figure S.2. SEM images and EDX mapping of KB50 (top) and KB25 (two bottom ones). EDX mapping indicate the presence of potassium, but extraction of an average particle size was not possible.

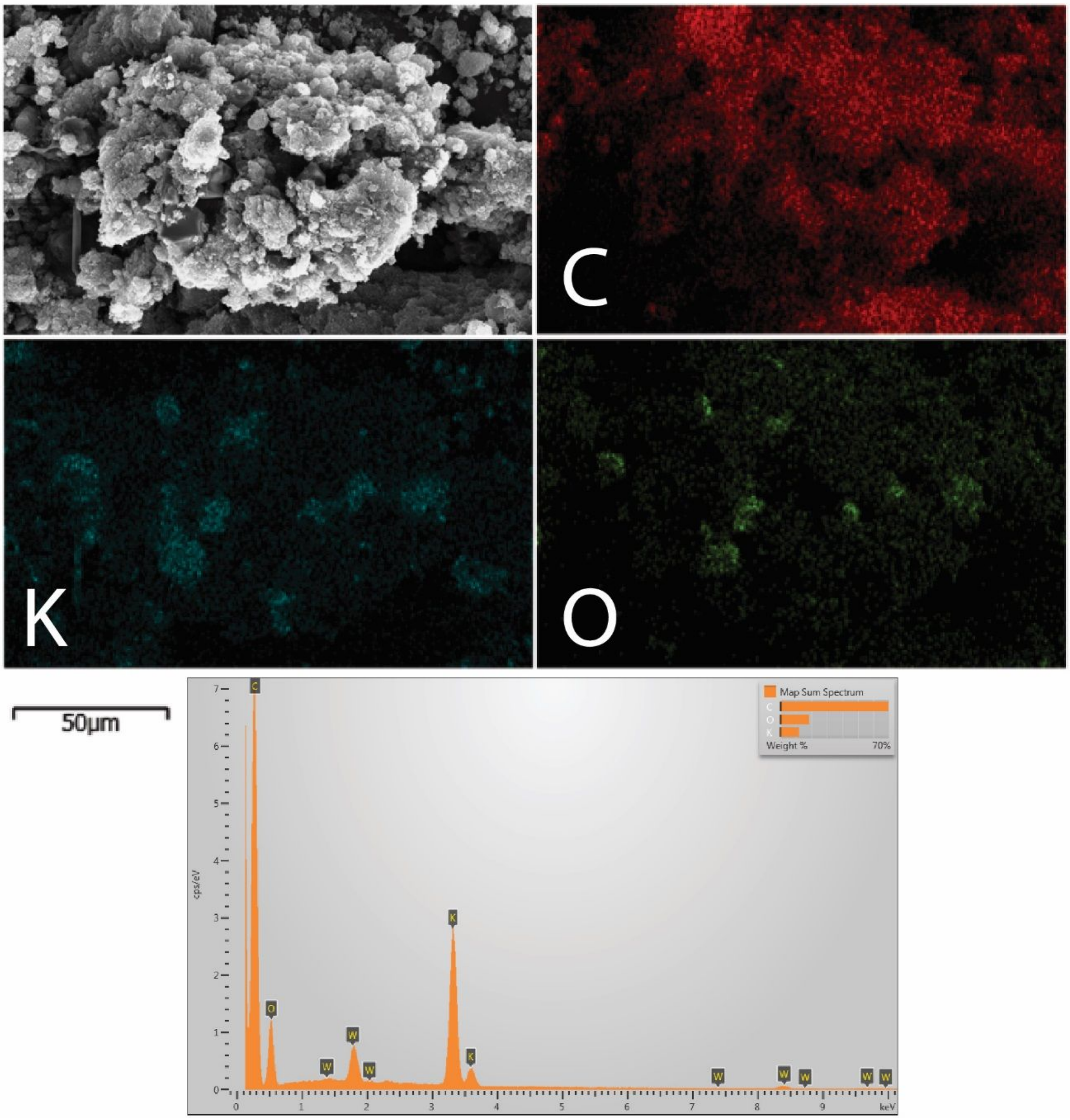

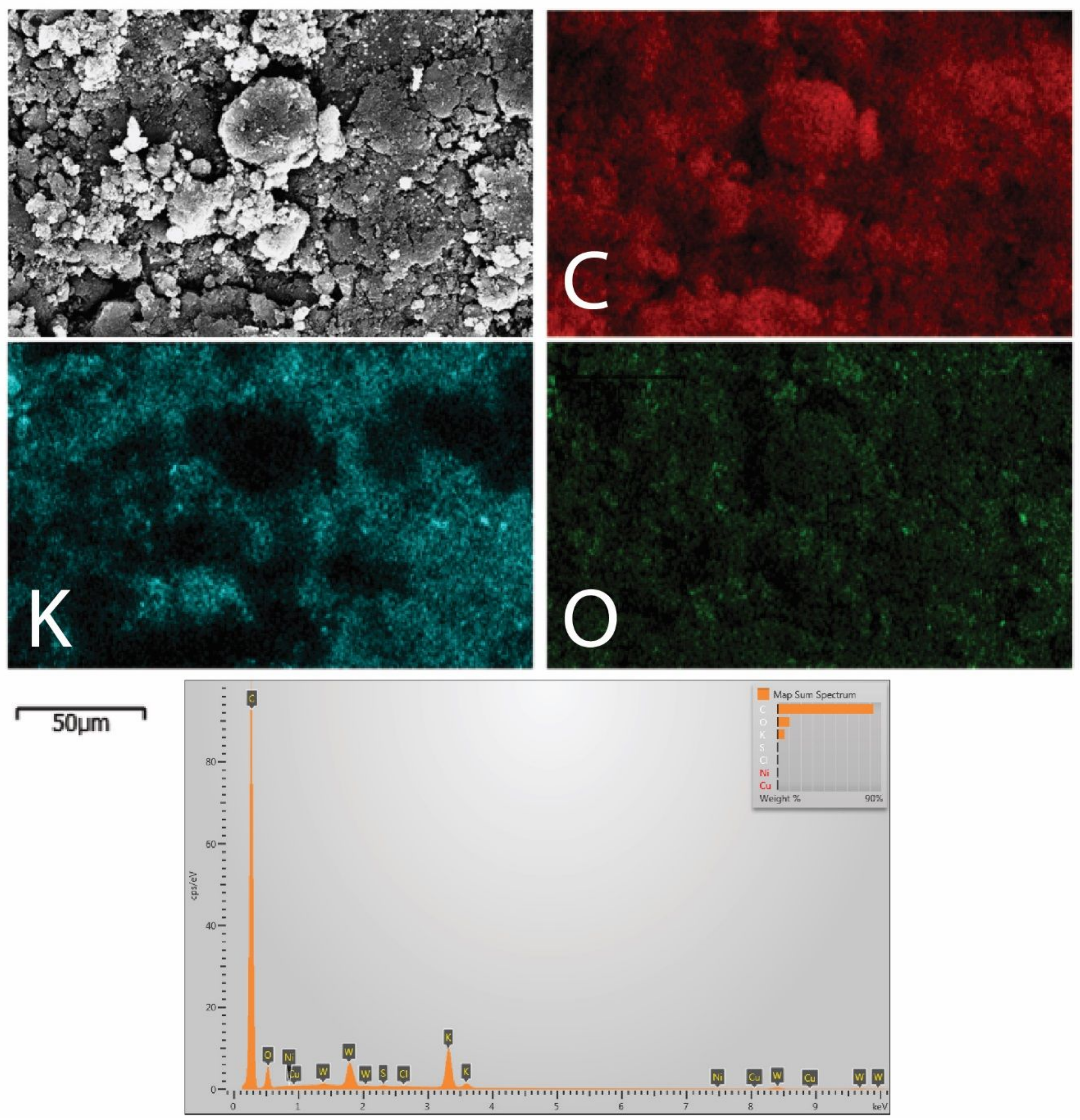
C
K
O
50µm
Map Sum Spectrum
C
O
K
S
Cl
Ni
Cu
Weight %
90%
cps/eV
keV

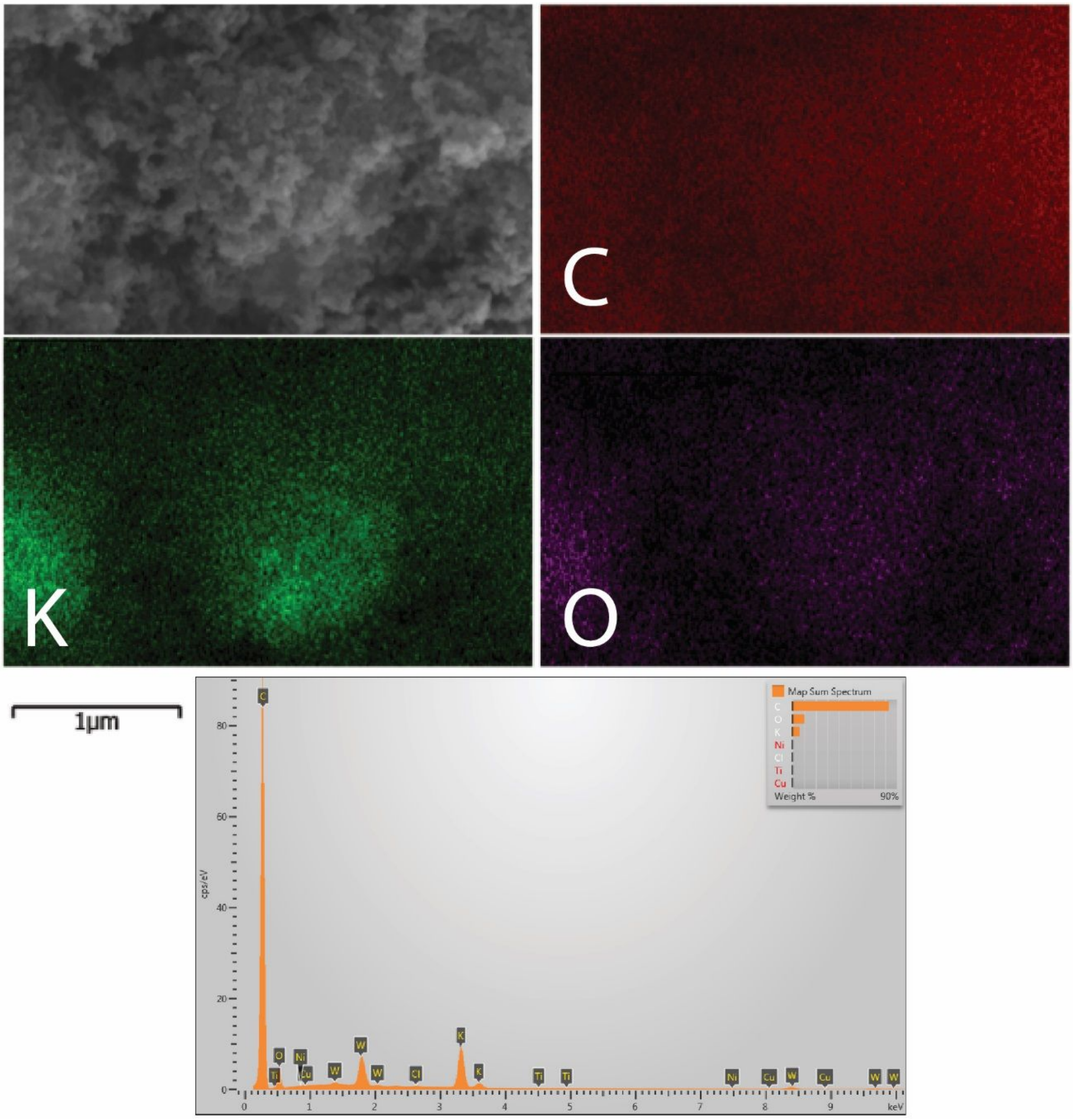
C
K
O
1µm
Map Sum Spectrum
C
O
K
Ni
Cl
Ti
Cu
Weight %
90%
cps/eV
keV

Note that background potassium is zero: SEM images and EDX mapping of the sample and an area on the sample holder. It shows that on the sample holder there is no background potassium.

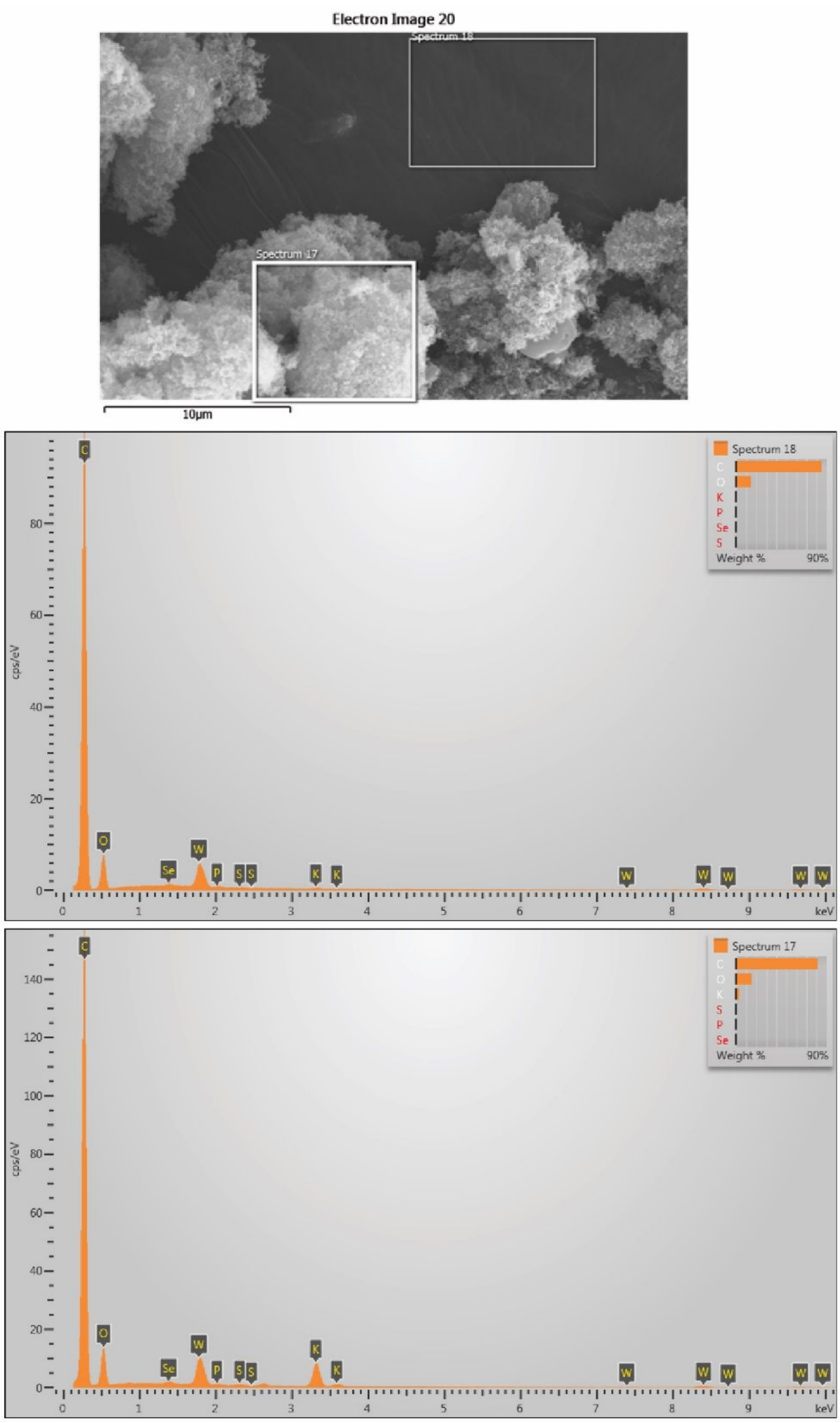

Figure S.3. TEM images of one K_2CO_3 particle captured over time. Sample (KB50) was exposed to beam (a). Images on the same particle were captured in sequence of 10 seconds (b, c, and d). Images show fast changes of the K_2CO_3 under the beam. The K_2CO_3 particles disappeared under the beam if the image is not captured fast enough.

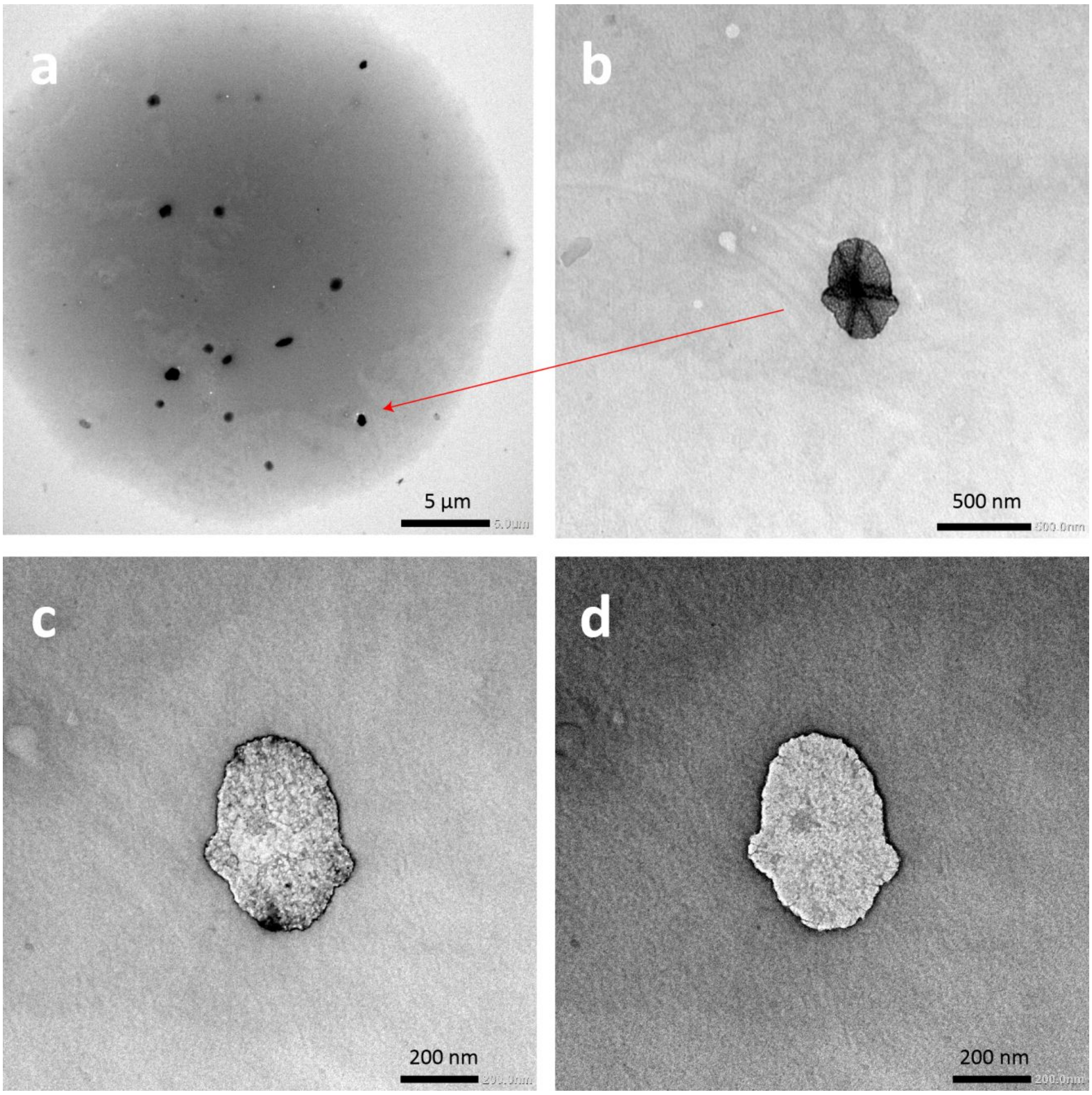

Quantitative XPS analysis

We used a model developed by Kerkhof and Moulijn[1] to estimate K_2CO_3 particle sizes for KB10, KB25, and KB50. The model suggests that a relative XPS intensity ratio sourced from an element from a metal or metal oxide to an element from a high surface area support relates to the dispersion of the metal/metal oxide on the support. The model assumes that the sample consists of sheets of support with cubic particles of the promoter (here we consider cubes of K_2CO_3 particles) with size of *c* in between support sheets (Scheme 1.a).

The thickness of the support sheets, *t*, is calculated as

$t = 2/\rho_s S_0$

where ρ_s is support density (carbon: 2.0 g/cm^3), and S_0 is the support surface area (for KetjenBlack: 1370 m^2/g) hence ***t* = 0.73 nm**.

It is assumed that electrons leave the sample only in a direction perpendicular to the surface. Using Lambert-Beer law, the model proposes formulas to calculate the amount of escaping electrons from one layer of the support ($I_{s,0}$) and one layer of the promoter ($I_{p,0}$):

$I_{s,0} = A\ n_s\ \sigma_s\ \lambda_{ss}\ (1 – exp\ (-t / \lambda_{ss}))$

$I_{p,0} = f\ A\ n_p\ \sigma_p\ \lambda_{pp}\ (1 – exp\ (-c / \lambda_{pp}))$

Where *A* is the effective area of the sample from which electrons reach the detector; the atomic densities in promoter and support (n_p, n_s); the photoelectron cross sections (σ_s, σ_p); the escape depths of the electrons (λ); and *f* is the fraction of support covered with promoter that follows:

$f = x / (1–x)\ S_0\ \rho_s\ c$

Here, *x* is the promoter weight fraction in the sample.

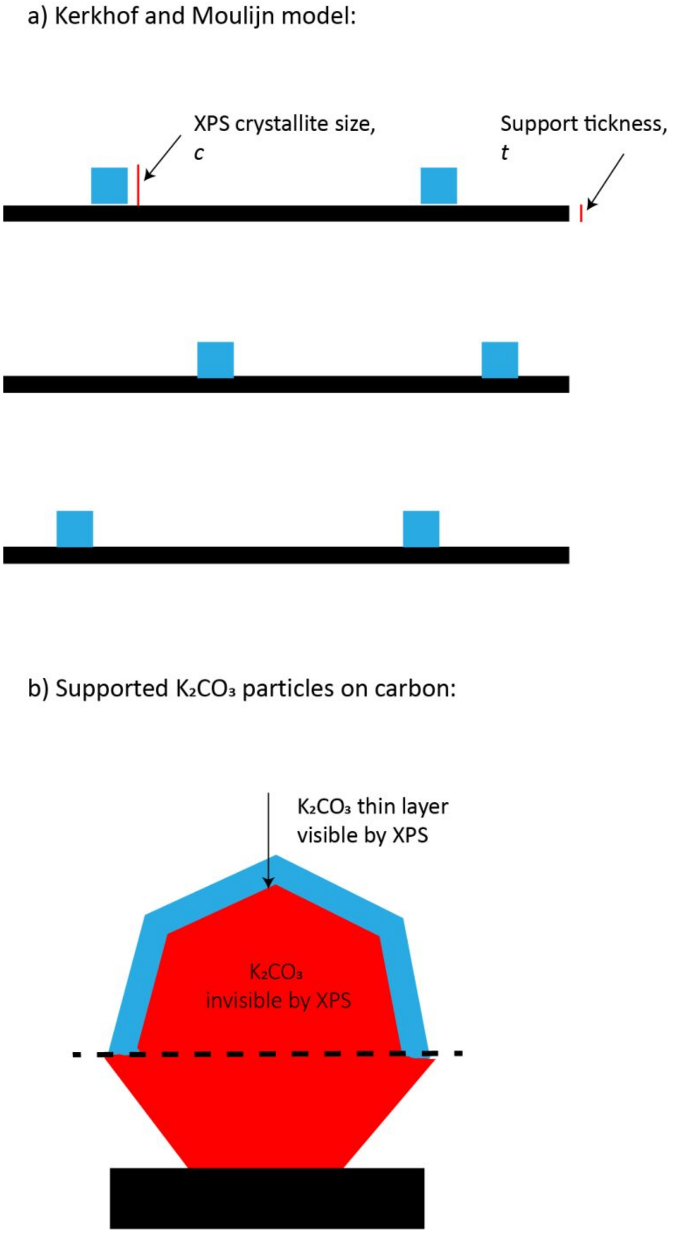

Scheme S.1. a) Schematic presentation of the sample described in the model and b) Supported K_2CO_3 particles on carbon: invisible and visible by XPS. In principal, the thickness of the blue layer should change because only perpendicular emission is assumed. Nevertheless, due to large size of particles compared to the penetration depth, this correction was waived.

Further, the total amount of escaping electrons from an infinite number of sheets is summed up considering that the most contribution to the signal is from the top layers. Samples with high surface area support like the KB series sorbents are assumed to have an infinite number of sheets. Hence, we use the model suggesting following equation for the experimentally obtained relative XPS intensities of promoter and support, $(I_p/I_s)_{exp}$. In our case, $(I_p/I_s)_{exp}$ is the relative XPS

intensities of potassium (K 2p) to carbon from support (C 1s, sp^2), and they are experimentally obtained from the high resolution K 2p and C 1s XP spectra of the samples (Figure S.3, Table S.1).

$$\left(\frac{I_p}{I_s}\right)_{exp} = \frac{D_{(\varepsilon p)}\, I_{p,0}\, (1 + P_{ps}\, P_{pp})(1 - P_{ss}\, P_{sp}^2)}{D_{(\varepsilon s)}\, I_{s,0}\, (1 - P_{ps}\, P_{pp}^2) P_{sp}}$$

D is detector efficiency as a function of the kinetic energy of the electrons. *P* is fraction of the electrons from one layer of promoter or support passing through another layer of promoter or support. The fraction of electrons from one support layer passing through a layer of support is P_{ss}, and the fraction from one layer of promoter passing through a layer of support P_{ps} and etc. They are functions of particle size, support thickness and support coverage (f):

$P_{ss} = exp\ (- \beta 1)$

$P_{ps} = exp\ (- \beta 2)$

$P_{sp} = 1 - f + f\ exp\ (- \alpha 2)$

$P_{pp} = 1 - f + f\ exp\ (- \alpha 1)$

Where dimensionless particle sizes are defined as:

$\alpha 1 = c/\ \lambda_{pp}$

$\alpha 2 = c/\ \lambda_{sp}$

And dimensionless support thickness are defined as:

$\beta 1 = t/\ \lambda_{ss}$

$\beta 2 = t/\ \lambda_{ps}$

Here,

λ_{pp} is the escape depth of electrons from the promoter traveling through the promoter,

λ_{ss} is the escape depth of electrons from the support traveling through the support,

λ_{sp} is the escape depth of electrons from the support traveling through the promoter, and

λ_{ps} is the escape depth of electrons from the promoter traveling through the support.

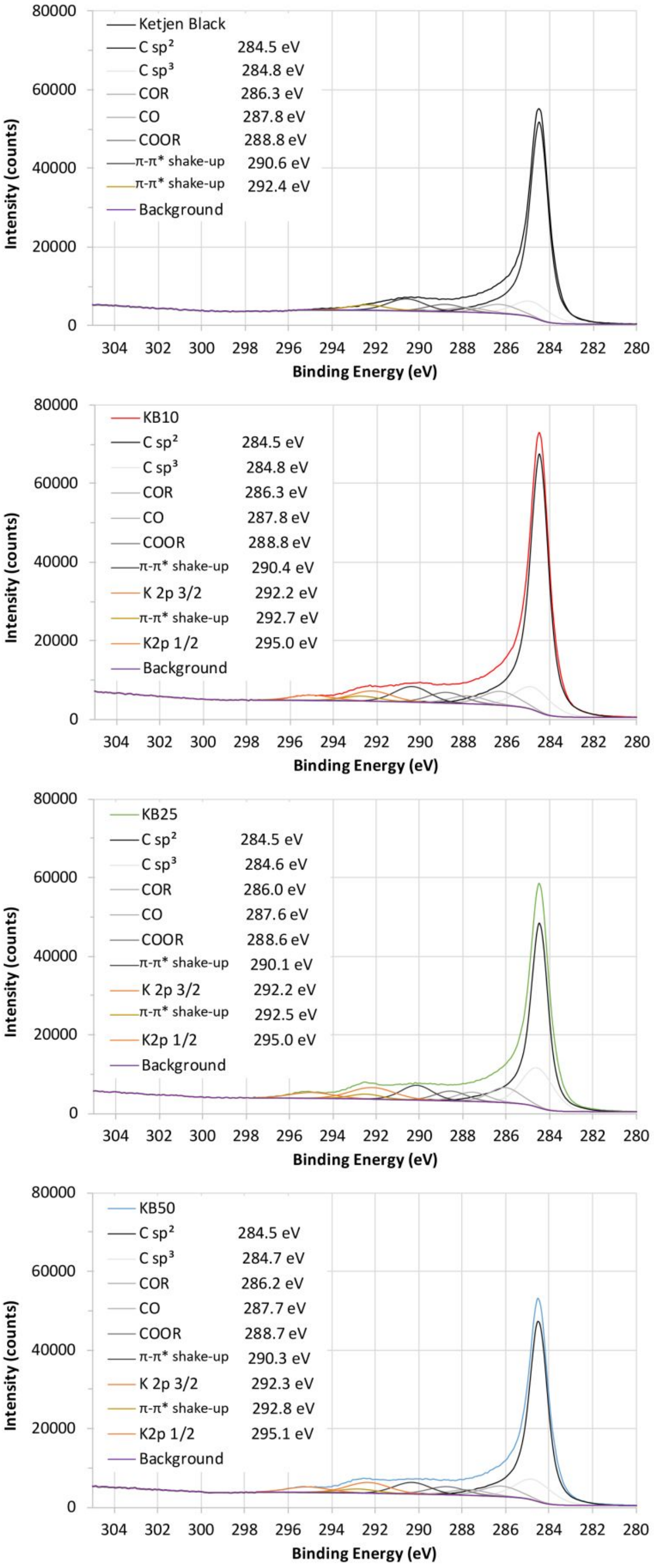

Figure S.4. High resolution K 2p and C1s XPS spectra of the KetjenBlack support, KB10, KB25, and KB50 samples

We try to simplify the formula to have a direct relation of the particle size to the experimentally obtained relative XPS intensities.

First, we assumed for our samples $P_{sp} \approx P_{pp} \approx 1$. It seems a valid assumption when *f*, coverage of support by the promoter, is negligible. We examined the validity of this assumption for two extreme cases of K_2CO_3 loadings and particle sizes on carbon considering support surface area is not lost during K_2CO_3 deposition:

1. For 10 wt.% K_2CO_3 on Ketjen Black with hypothetical cubic particles of K_2CO_3 with size of c=10 nm, *f* is 0.033
2. For 50 wt.% K_2CO_3 on Ketjen Black with hypothetical cubic particles of K_2CO_3 with size of c=50 nm, *f* is 0.006

To experimentally confirm this assumption, we looked at the intensity of C 1s, sp^2, i.e. the carbon signal sourced from the Ketjen Black support, in Ketjen Black and KB50 spectra (Figure S.3). XPS spectra show that the intensity of C 1s, sp^2 in KB50 is not diminished compared to the intensity of C 1s, sp^2 in Ketjen Black. Hence, we at least experimentally observed that $P_{sp} \approx 1$.

Hence the formula simplifies to:

$$\left(\frac{I_p}{I_s}\right)_{exp} = \frac{D_{(\varepsilon p)}\, I_{p,0}\, (1 + P_{ps})(1 - P_{ss})}{D_{(\varepsilon s)}\, I_{s,0}\, (1 - P_{ps})}$$

By substituting $I_{p,0}$, $I_{s,0}$, and the bulk atomic ratio of the support and the promoter $(p/s)_b$:

$(p/s)_b = x\, n_p\, \sigma_s / (1\text{–}x)\, n_s\, \sigma_p$,

the equation is elaborated to:

$$\left(\frac{I_p}{I_s}\right)_{exp} = \left(\frac{p}{s}\right)_b \frac{D_{(\varepsilon p)}\, \sigma_p\, (1 - \exp(-\alpha_1))\, \beta_1\, (1 + \exp(-\beta_2))}{D_{(\varepsilon s)}\, \sigma_s \qquad \alpha_1 \qquad 2\, (1 - \exp(-\beta_2))}$$

When electrons with small differences in kinetic energy are studied like electrons of K2p and C1s, both at binding energies of ca. 290 eV (at constant $h\nu$ = 1486.6 eV), $D_{(\varepsilon p)} = D_{(\varepsilon s)}$, $\lambda_{ps} = \lambda_{ss}$, and consequently $\beta 1 = \beta 2 = \beta$

β is extracted by calculation of the λ_{ss}, the escape depth of electrons from the support traveling through the support. The escape depths of the electrons for C 1s was calculated as 3.67 nm in carbon media at 1486.6 eV photon energy. The escape depths of the electrons were extracted from NIST Electron Inelastic-Mean-Free-Path Database, software package version 1.2, according to predictive formulae of TPP-2M equation of Tanama, Powell and Penn.[2] Since ***t* is 0.73**, ***β* is 0.2**.

The equation is reduced to

$$\left(\frac{I_p}{I_s}\right)_{exp} = \left(\frac{p}{s}\right)_b \frac{\sigma_p\, (1 - \exp(-\alpha_1))}{\sigma_s \qquad \alpha_1}$$

Here, α_1 contains particle size information and is obtained as follows.

For a hypothetical monolayer of promoter on the support, α is small and $(1 - exp(-\alpha_1))/\alpha_1$ goes to 1. Hence hypothetical $(I_p/I_s)_{monolayer}$ is equal to $(p/s)_b\, (\sigma_p/\sigma_s)$ and needs to be calculated for KB50, KB25, and KB10 samples. The bulk atomic ratio of the support and the promoter $(p/s)_b$ is known for these three samples (Table S.1). The photoelectron cross sections for C1s and K 2p were extracted from calculations of Scofield[3] at a

photon energy of 1486.6 eV. The photoelectron cross section for C1s is 1, for K $2p_{1/2}$ is 1.35 and for K $2p_{3/2}$ is 2.62. In total σ_p/σ_s = (1.325+2.62)/1 = 3.97.

Further, the percentage of the monolayer is calculated as $(I_p/I_s)_{exp}$ / $(I_p/I_s)_{monolayer}$. This percentage is equal to $(1 - exp(-\alpha_1))/\alpha_1$. From a graph (Figure S.4) that relates $(1 - exp(-\alpha_1))/\alpha_1$ to α_1, corresponding α_1 is obtained for KB50, KB25, and KB10 samples (Table S.1).

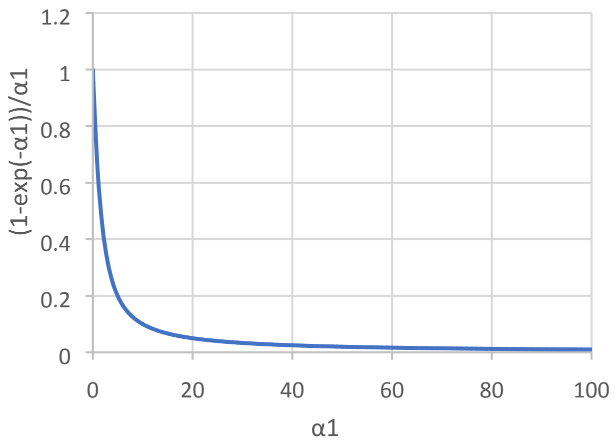

Figure S.5. General relation of ***(1 – exp (-α1))/α1*** to ***α1*** showing the percentage of a monolayer promoter on the support

The escape depths of the electrons for K 2p is calculated as 3.3 nm in K_2CO_3 media at 1486.6 eV photon energy. The escape depths of the electrons were calculated with NIST Electron Inelastic-Mean-Free-Path Database, software package version 1.2, according to predictive formulae of TPP-2M equation of Tanuama, Powell and Penn.[2] The TPP-2M equation is a function of electron energy (1486.6 eV), density (2.43 g/cm^3), number of valence electrons per atom (24), and the bandgap energy (3.697 eV)[4] of K_2CO_3. Accordingly, particle sizes for KB50, KB25, and KB10 are calculated as shown in the table.

Table S.1. Summarized variables applied in the model and the model prediction for K_2CO_3 particle sizes

Sample	K (mol)	C from support (mol)	K/C (mol/mol)	*Calculated* $(I_p/I_s)_{monolayer}$	$(I_p/I_s)_{exp}$	Monolayer percentage (%)	α_1	Estimated particle size by the model (nm)
**KB10**	0.14	7.50	0.02	0.05	0.051 ± 0.015	66	0.88	3 ± 2
**KB25**	0.36	6.25	0.06	0.15	0.096 ± 0.027	42	2.11	7 ± 3
**KB50**	0.72	4.17	0.17	0.46	0.077 ± 0.022	8	9.00	30 ± 8

Figure S.6. TGA on KetjenBlack support, bulk K_2CO_3, KB10, KB25, and KB50 samples. Samples heated up to 920 °C under flow of N_2 (50 mL/min). Under flow of nitrogen, oxidation of carbon occurred at temperatures around the melting point of the K_2CO_3. No meaningful heat flow could be attributed to the K_2CO_3 melting. Hence, air was used instead of inert N_2 . According to thermodynamic calculations by HSC software, the reduction of K_2CO_3 to gaseous potassium takes place at the presence of carbon as a reducing agent at temperatures above 900 °C (Figure S.7). This reaction and the heat flow associated with that eclipsed the melting of the K_2CO_3.

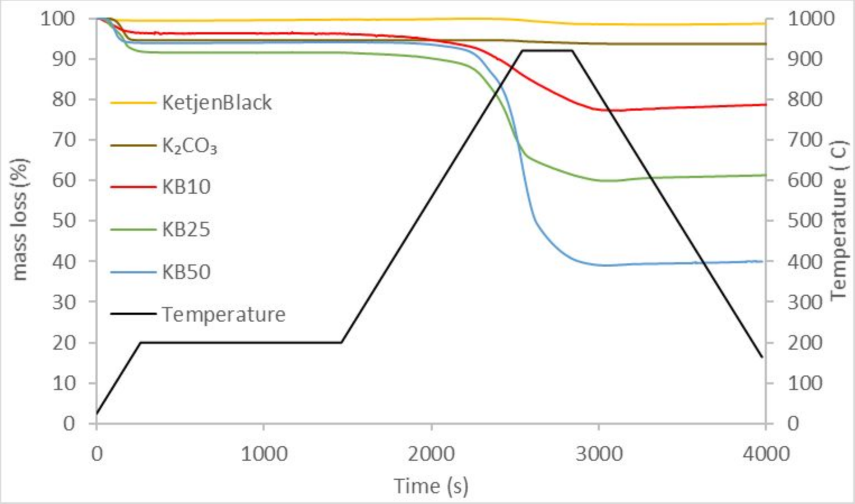

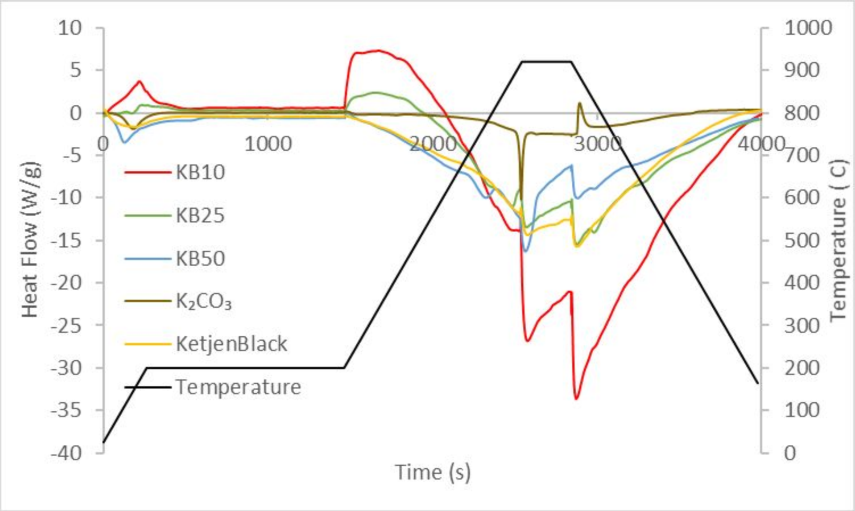

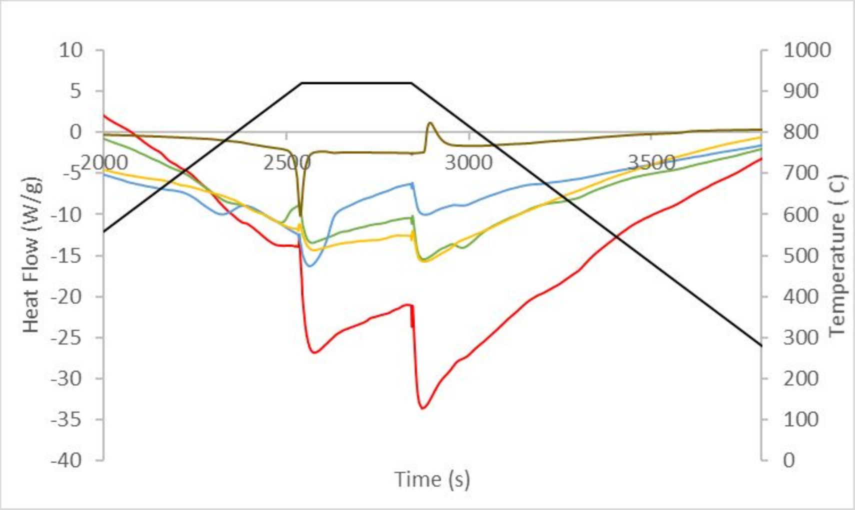

Figure S.7. Thermodynamically stable components under nitrogen at high temperatures obtained by thermodynamic database of the HSC 8 software

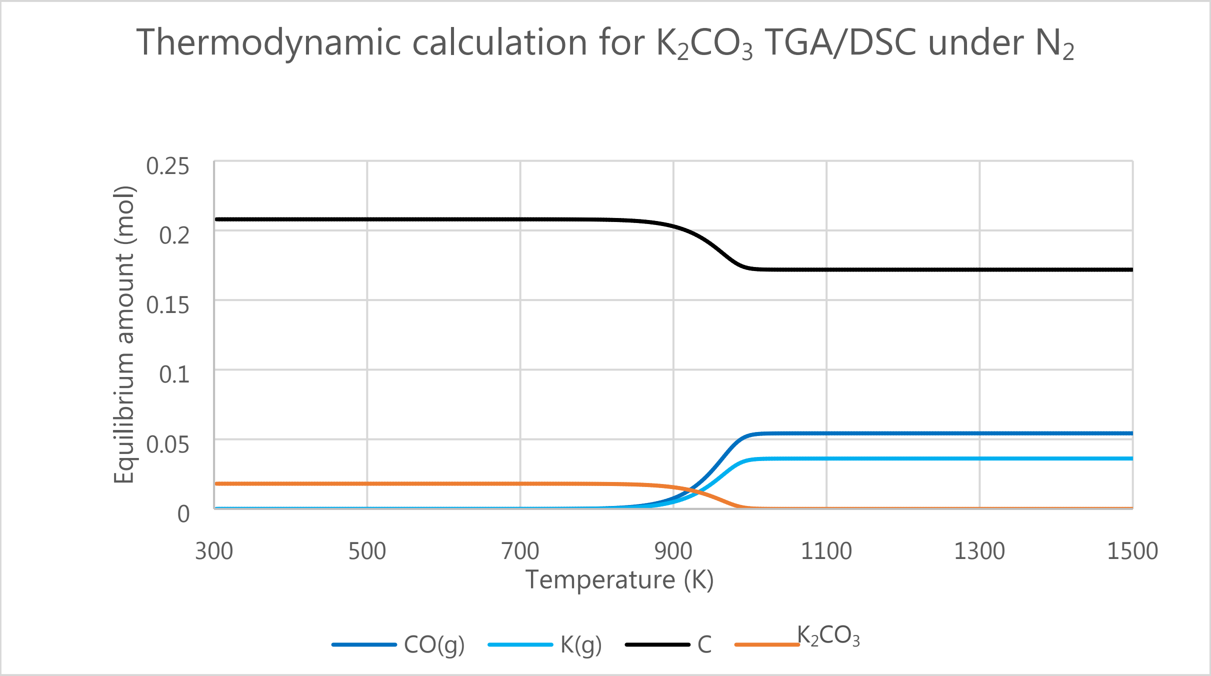

Figure S.8. The relation of the melting point to the K_2CO_3 particle size according to the Gibbs-Thomson equation. At temperatures higher than 880 °C, slight variation in melting point leads to a considerable change in particle diameter.

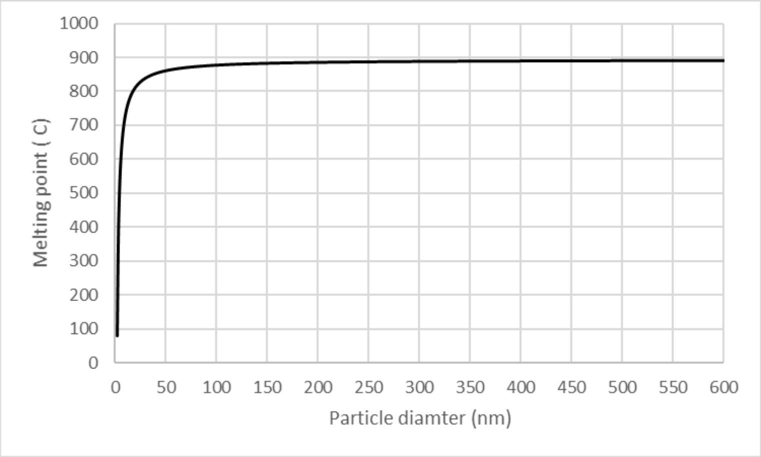

Figure S.9. TGA mass increase upon exposure to wet air. Bulk K_2CO_3 physically mixed with the Ketjen Black (around 2 mg of sample), Pre-treated at 200 °C under 150 mL/min flow of dry synthetic air (that did not contain CO_2) for 1 hour, Adsorption at 60 °C under 150 mL/min flow of air that contained different amount of water for 3 hours. Post treatment at 200 °C under 150 mL/min flow of dry synthetic air for 1 hour.

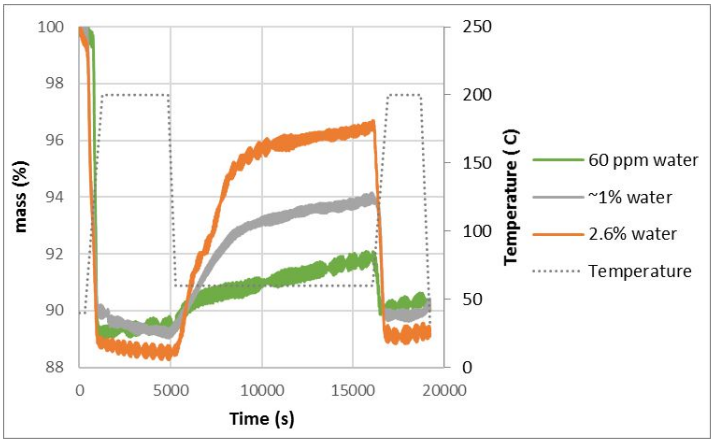

The figure shows the mass increase upon exposure of the bulk K_2CO_3 (K_2CO_3 which physically mixed in a mortar with the Ketjen Black support) to a wet air flow with different water content during TGA. More water content of the flow led to a more calculated CO_2 uptake. We suspect that the CO_2 uptake measured by TGA is subject to error due to interfering water uptake that overestimates the CO_2 uptake. On the other hand, water concentration may affect the sorption of CO_2 and/or water. These two effects are not easily distinguishable. Hence kinetics reported under TGA condition is not representative of the kinetics of the reaction in a flow reactor.

Table. CO_2 sorption capacity of the bulk K_2CO_3 under different amount of water

	Mass increase upon exposure to air (%)	Calculated CO_2 uptake (mmol CO_2/g sorbent)	CO_2 uptake (mol CO_2/mol K_2CO_3)
60 ppm water	2.45	0.35	0.10
~1 % water	4.76	0.67	0.19
2.6 % water	9.01	1.27	0.36

CO_2 uptake is measured by weight increase upon exposure to air assuming 1:1 stoichiometric ratio of water and CO_2 uptake

Figure S.10. TGA on KB10, KB25, and KB50 samples and corresponding heat flows during decarbonation. Samples that were stored in atmospheric conditions were heated up to high temperatures under flow of synthetic air (50 mL/min, zero CO_2). The temperature of decarbonation during TGA matches the decarbonation temperature in the flow unit.

The enthalpy of decarbonation was calculated as 120 kJ/mol. It is more than reported value of 107 kJ/mol.[5] CO_2 uptake was also more than 1 mol/mol K_2CO_3. It means part of the mass loss is due to dehydration instead of decarbonation.

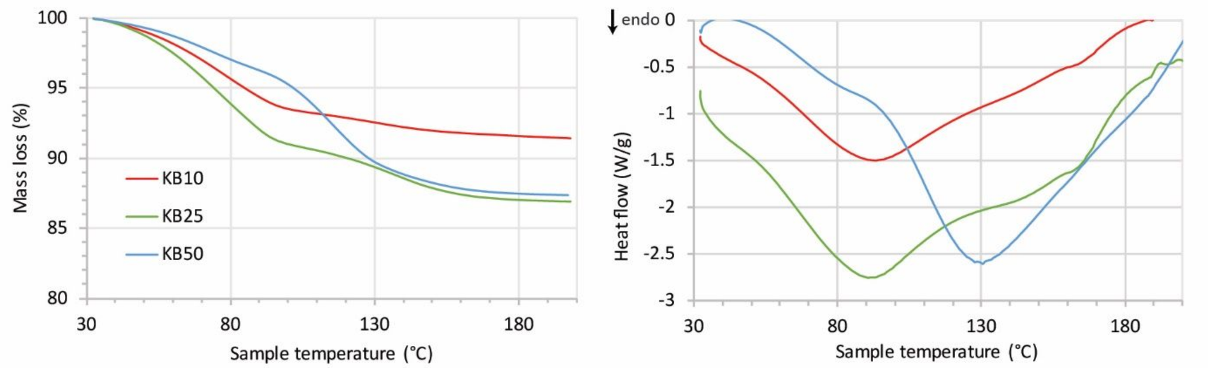

Details of the calculation of the apparent activation energy for decarbonation steps

Redhead analysis[6] provides a simple way for the estimation of apparent activation energies for a first order desorption step in a typical temperature programmed desorption measurement. The analysis is based on Arrhenius equation and assumes the apparent activation energy of desorption (E_d) and pre-exponential factor (A) are coverage independent.

The analysis suggests the following equation to relate the heating rate (β) to the temperature that a maximum desorption peak (T_p) appears,

$$\frac{E_d}{R\,T_p^2} = \frac{A}{\beta}\exp\left(\frac{-E_d}{R\,T_p}\right)$$

Where R is the gas constant. By rearranging the formula the following is obtained,

$$Ln\frac{\beta}{T_p^2} = \frac{-E_d}{R\,T_p} + \frac{A\,R}{E_d}$$

Hence, by plotting the $Ln\frac{\beta}{T_p^2}$ vs $\frac{1}{Tp}$, (below figure), where T_p is the temperature of the first and second decarbonation steps, we obtained the apparent activation energies for both decarbonation steps as 69 kJ/mol and 93 kJ/mol.

Table S.2. Variables of the Redhead analysis from the decarbonation upon heat treatment data

Heating rate, β (°C/min)	Temperature of first decarbonation step, T_p1 (K)	Temperature of second decarbonation step, T_p2 (K)
5	377	413
10	389	420
20	400	433

Figure S.11. Arrhenius plot of the decarbonation steps

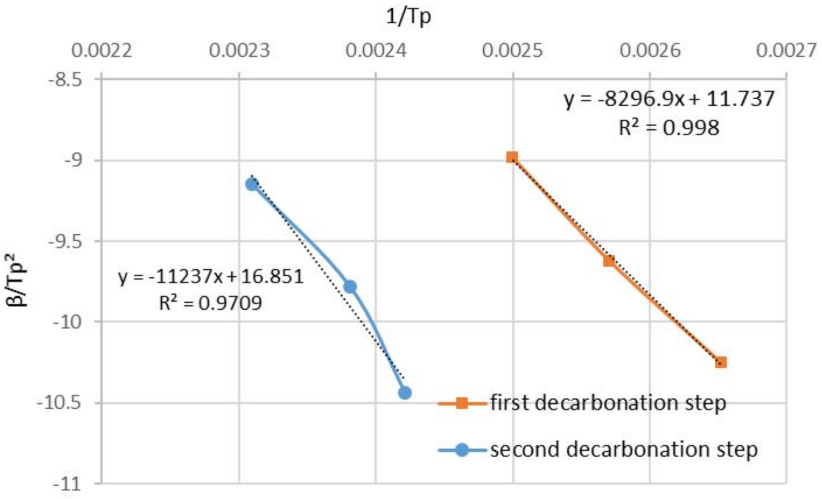

References

(1) Kerkhof, F. P. J. M.; Moulijn, J. A. Quantitative analysis of XPS intensities for supported catalysts. *The Journal of Physical Chemistry* **1979,** *83* (12), 1612.

(2) Powell, C. J.; Jablonski, A. *NIST Electron Inelastic-Mean-Free-Path Database - Version 1.2, SRD 71, National Institute of Standards and Technology, Gaithersburg, MD*, 2010.

(3) Scofield, J. H. Hartree-Slater subshell photoionization cross-sections at 1254 and 1487 eV. *Journal of Electron Spectroscopy and Related Phenomena* **1976,** *8* (2), 129.

(4) Duan, Y.; Luebke, D. R.; Pennline, H. W.; Li, B.; Janik, M. J.; Halley, J. W. Ab Initio Thermodynamic Study of the CO2Capture Properties of Potassium Carbonate Sesquihydrate, K2CO3·1.5H2O. *The Journal of Physical Chemistry C* **2012,** *116* (27), 14461.

(5) Meis, N. N. A. H.; Frey, A. M.; Bitter, J. H.; de Jong, K. P. Carbon Nanofiber-Supported K2CO3 as an Efficient Low-Temperature Regenerable CO2 Sorbent for Post-Combustion Capture. *Industrial & Engineering Chemistry Research* **2013,** *52* (36), 12812.

(6) Redhead, P. A. Thermal desorption of gases. *Vacuum* **1962,** *12* (4), 203.
